# The epidemiology of hepatitis C virus in Central Asia: Systematic review, meta-analyses, and meta-regression analyses

**DOI:** 10.1038/s41598-019-38853-8

**Published:** 2019-02-14

**Authors:** Welathanthrige S. P. Botheju, Fawzi Zghyer, Sarwat Mahmud, Assel Terlikbayeva, Nabila El-Bassel, Laith J. Abu-Raddad

**Affiliations:** 1 Weill Cornell Medicine - Qatar, Cornell University, Qatar Foundation - Education City, Doha, Qatar; 2 Infectious Disease Epidemiology Group, Weill Cornell Medicine - Qatar, Cornell University, Qatar Foundation - Education City, Doha, Qatar; 3 Global Health Research Center of Central Asia in Kazakhstan, Almaty, Kazakhstan; 4 Social Intervention Group, Columbia University School of Social Work, New York, New York USA; 5 Department of Healthcare Policy and Research, Weill Cornell Medicine, Cornell University, New York, New York USA; 6 College of Health and Life Sciences, Hamad bin Khalifa University, Doha, Qatar

## Abstract

The objective was to delineate hepatitis C virus (HCV) epidemiology in countries of Central Asia (CA), specifically Kazakhstan, Kyrgyzstan, Tajikistan, Turkmenistan, and Uzbekistan. A systematic review was conducted guided by the Cochrane Collaboration Handbook, and reported using PRISMA guidelines. Meta-analyses were performed using DerSimonian-Laird random-effects models with inverse variance weighting. Random-effects meta-regression analyses were performed on general population studies. The systematic review identified a total of 208 HCV prevalence measures. No incidence or Turkmenistan studies were identified. Meta-analyses estimated HCV prevalence among the general population at 0.7% (95%CI: 0.7–0.8%) in Kazakhstan, 2.0% (95%CI: 1.7–2.4%) in Kyrgyzstan, 2.6% (95%CI: 1.7–3.6%) in Tajikistan, and 9.6 (95%CI: 5.8–14.2%) in Uzbekistan. Across CA, the pooled mean prevalence was 13.5% (95%CI: 10.9–16.4%) among non-specific clinical populations, 31.6% (95%CI: 25.8–37.7%) among populations with liver-related conditions, and 51.3% (95%CI: 46.9–55.6%) among people who inject drugs. Genotypes 1 (52.6%) and 3 (38.0%) were most frequent. Evidence was found for statistically-significant differences in prevalence by country, but not for a temporal decline in prevalence. CA is one of the most affected regions by HCV infection with Uzbekistan enduring one of the highest prevalence levels worldwide. Ongoing HCV transmission seems to be driven by injecting drug use and healthcare exposures.

## Introduction

With approximately 71 million people chronically infected worldwide, hepatitis C virus (HCV) related morbidities place a strain on healthcare systems globally^1^. Since the recent development of direct-acting antivirals (DAA), a breakthrough treatment which provides opportunities to reduce HCV infection and disease burden^2,3^, the World Health Organization (WHO) has set a target for the elimination of HCV as a public health concern by 2030^4,5^. As such, an understanding of HCV epidemiology and risk factors for HCV infection worldwide is essential for developing targeted and cost-effective preventative and treatment interventions, to achieve the global target and eliminate HCV.

Geographically, for the purpose of this study, Central Asia (CA) encompasses five countries: Kazakhstan, Kyrgyzstan, Tajikistan, Turkmenistan, and Uzbekistan. Since independence from the Soviet Union, these countries have been undergoing difficult political, social, and economic transition^6,7^. The public health and healthcare infrastructure has deteriorated, resulting in a decline in life expectancy, a rising burden of diseases, and re-emergence of infectious diseases^7,8^. Though the region is perceived to have one of the highest HCV prevalence levels worldwide^9,10^, HCV epidemiology and the drivers of HCV transmission remain poorly characterized.

Our objective was to delineate HCV epidemiology in CA by (1) performing a systematic review of all available records of HCV antibody incidence and/or antibody prevalence among the different population categories, (2) pooling all HCV antibody prevalence measures in the general population to estimate the country-specific population-level HCV prevalence, (3) estimating the number of HCV infected persons across countries of CA, (4) performing a secondary systematic review of all evidence on HCV genotype information, and (5) identifying sources of between-study heterogeneity and estimate their contribution to the variability in HCV prevalence among the general population.

## Materials and Methods

The methodology in this study is informed and adapted from that of the systematic reviews of the Middle East and North Africa (MENA) HCV Epidemiology Synthesis Project^11–19^. This methodology is summarized in the ensuing subsections, and additional information is available in respective publications from this project^11–19^.

### Sources of data and search strategy

Literature on HCV antibody incidence and/or antibody prevalence was systematically reviewed guided by the Cochrane Collaboration Handbook^20^. Preferred Reporting Items for Systematic Reviews and Meta-Analyses (PRISMA) guidelines were used in reporting our results^21^ (Table S1). The data sources used in this study included international PubMed and EMBASE databases (up to 9^th^ April, 2018), a Russian scientific database—Scientific Electronic Library (eLibrary.ru) (up to 9^th^ April, 2018), and country-level reports. The search criteria was broad with no language restrictions (Fig. S1). Articles published after 1989 were included in this review, since this was the year in which HCV was first identified^22,23^.

### Selection of studies

Duplicate publications were found and removed using the reference manager software, Endnote. Screening of the remaining unique records’ titles and abstracts were performed individually by WB and FZ. Articles that were considered relevant or potentially relevant underwent full-text screening, using our inclusion and exclusion criteria. The references of all full-text articles and literature reviews also underwent screening to find any further relevant articles that may have been overlooked.

### Inclusion and exclusion criteria

The inclusion and exclusion criteria used were adapted from that of the MENA HCV Epidemiology Synthesis Project systematic reviews^11–19^. The inclusion criteria consisted of any document reporting HCV antibody incidence and/or antibody prevalence in populations from Kazakhstan, Kyrgyzstan, Tajikistan, Turkmenistan, and Uzbekistan, based on primary data, and of any language. The exclusion criteria included studies conducted before 1989, studies that referred to HCV as non-A non-B hepatitis, case series, case reports, commentaries, editorials, letters to editors, and literature reviews. All records underwent a secondary independent screening for data on HCV genotypes, regardless of whether they reported HCV antibody incidence and/or antibody prevalence.

In the following subsections, the term ‘report’ is used to refer to any document with an outcome measure of interest, while a ‘study’ refers to stratifications of a specific outcome measure. As such, a single report may contribute multiple studies, and multiple reports of the same study (outcome measure) were recognized as duplicates and considered as one study.

### Extraction and analyses of data

**Figure 1:**
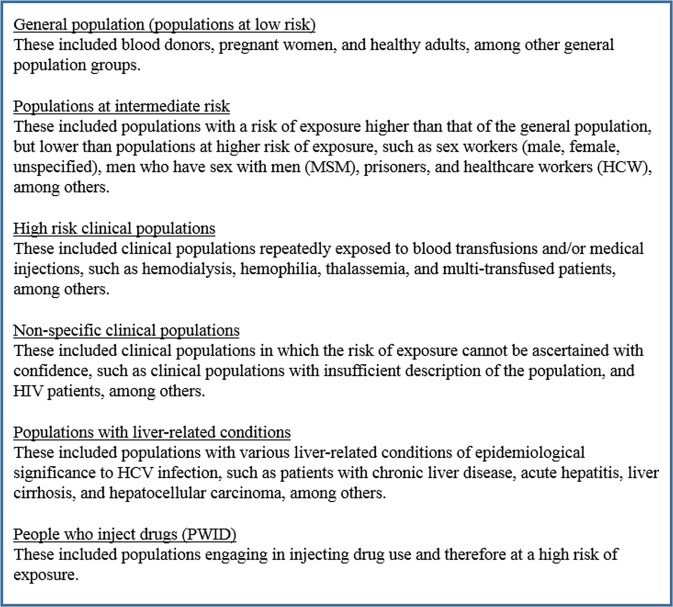
Population classification into categories by risk of exposures to hepatitis C virus (HCV) infection.

Data from all reports considered relevant were extracted by WB and FZ. Data from all reports were subsequently double extracted by SM to ensure consistency and minimize errors in extracted information. Extracted information included study details (author, year of publication, title, and journal), location of study, year(s) of data collection, study design, sampling method, risk population, number of participants included in the study, number of participants tested, type and name of serological test used to test for HCV, and the primary outcome (HCV incidence or/and HCV prevalence). Rounding HCV prevalence measures to two decimal places was conducted if they were below 1%, while the remaining measures were rounded to only one decimal place. When available, HCV ribonucleic acid (RNA) data were also extracted. All studies identified in the secondary independent screening for genotype information were also extracted into a separate extraction file. Risk factors for HCV infection were extracted if they were statistically-significant through multivariable meta-regression. Extracted data were classified into population categories according to exposure risk to HCV infection, as presented in Fig. 1. The classification scheme was based on existing literature^10,24,25^, and earlier reviews of HCV prevalence^11–19^.

### Quantitative assessment

HCV prevalence reports with a minimum of 50 participants were categorized and reported in our reporting tables by risk population. Meta-analyses of HCV prevalence measures were performed by risk population and country for all studies with at least 25 participants. In reports where HCV prevalence was reported for mixed-country samples, the study was included only in meta-analyses for CA as a region. In reports that included prevalence measures but no reported sample size, a sample size of 300 was imputed and the study was included in the review and meta-analyses. This sample size was deemed reasonable and conservative, given that the median sample size of included studies with a reported sample size was 348.

HCV prevalence for the total sample size was replaced with stratified prevalence whenever a minimum of 25 participants were available for each stratum. Stratifications were included based on a predefined order, where nationality was prioritized, then sex, year, region, and finally age. To avoid duplication one final stratification for each study was included.

Freeman-Tukey type arcsine square-root transformation was used to stabilize the variance of HCV prevalence measures^26^. DerSimonian-Laird random-effects model was used to pool HCV prevalence (with inverse variance weighting). This model assumes a normal distribution of true effect sizes (that is HCV prevalence) across studies, and takes into account true heterogeneity as well as random chance effects across studies^27^.

Heterogeneity measures were also assessed. All forest plots were visually assessed and the Cochran’s Q test was performed, with a p-value of <0.10 indicating statistically strong evidence^27,28^. The I² measure and its confidence interval were assessed^27^. The prediction interval was also calculated to estimate the range in which HCV prevalence of 95% of future studies will fall^27,29^.

The number of HCV antibody-positive persons in each country was determined by multiplying the country-specific pooled mean HCV antibody prevalence estimate by the population size in each country. This was subsequently multiplied by the pooled mean fraction of HCV RNA positivity in antibody-positive persons (also commonly referred to as the “viremic rate”^30,31^), to derive the number of HCV chronically-infected persons. The United Nations World Population Prospects database^32^ was used to obtain the population size of each country.

Since potential issues have been identified with the Freeman-Tukey type arcsine square-root transformation^33^, a sensitivity analyses was performed to confirm the validity of our results in which the generalized linear mixed models (GLMM) method was used to perform meta-analyses.

A proportion of the general population data were on blood donors, a population typically including only healthy adults. Sensitivity analysis was performed to determine whether excluding blood donors could impact the pooled mean HCV prevalence estimate in the general population. This sensitivity analysis was done for each country separately, and for CA as a whole.

Based on established methodology^20^, univariable and multivariable random-effects meta-egressions were performed to assess country-level associations with HCV prevalence and the sources of between-study heterogeneity in the general population. Variables included in the univariable models included country, general population subpopulations, study site, sample size (<100 or ≥100), sampling method (probability-based or non-probability-based), year of publication, and year of data collection. Variables with a p-value of <0.1 were included in the multivariable model. Variables were deemed significant in the final multivariable meta-regression if they had a p-value of <0.05.

For each country and the whole CA, the frequency of each genotype was calculated. Individuals who were positive for mixed genotypes contributed separately to the number of each of the identified genotypes. The Shannon Diversity Index (H) was determined to assess the diversity of genotypes, with a higher score (out of 1.95) indicating more diversity^34^.

The *meta* package^35^ on R version 3.4.3^36^ was used to perform the meta-analyses. The *metan* command on STATA 13^37^ was used to perform meta-regressions.

### Qualitative analysis

Using the Cochrane approach to surmise risk of bias (ROB), the quality of HCV incidence and/or prevalence measures was evaluated. Based on three quality domains, studies were classified into either *low* or *high* ROB. These domains included HCV ascertainment (biological assay or otherwise), sampling method (probability-based or non-probability-based), and response rate (≥80% of the target sample size was reached or otherwise).

Studies with information missing for any of the three domains were classified with *unclear* ROB for that specific domain. Studies in which the reported HCV measures were acquired from patients’ medical records, or from individuals voluntarily visiting facilities where routine blood screening is performed, were considered as having *low* ROB on strictly the response rate domain.

Studies with at least 100 participants were classified as having *high* precision, as informed by previous studies^11–19^.

## Results

### Search results

**Figure 2:**
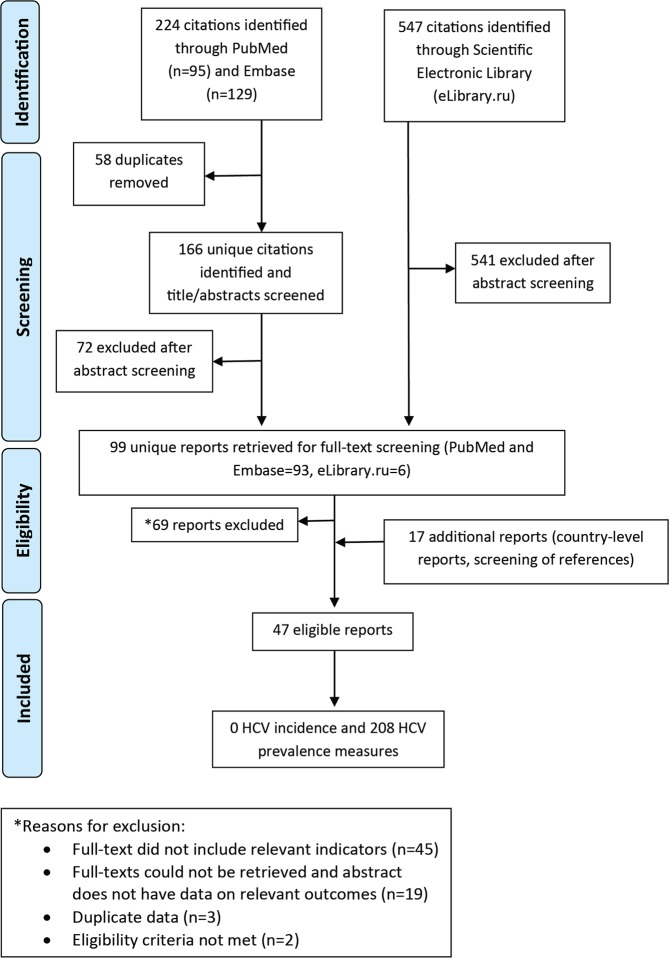
Flow chart of the process by which articles were selected for inclusion in this systematic review of hepatitis C virus (HCV) incidence and prevalence in Central Asia, adapted from the Preferred Reporting Items for Systematic Reviews and Meta-Analyses (PRISMA) 2009 guidelines^21^.

Figure 2, adapting the PRISMA flow diagram^21^, shows the process by which studies were selected into this systematic review. A total of 771 citations were identified: 95 from PubMed, 129 from Embase, and 547 from the Scientific Electronic Library (eLibrary.ru). A total of 99 unique reports underwent full-text screening, after duplicates were removed and titles and abstracts were screened. From these, 69 reports were removed, the reasons for which are stated in Fig. 2. Eighteen reports were added to the systematic review from gray literature/unpublished data, and from screening of references of full-text articles and reviews. Finally, 47 reports qualified for inclusion in this systematic review, yielding no incidence measure and 208 prevalence measures.

In the secondary systematic review, all 771 citations were screened for HCV genotype information. After duplicates were removed and titles and abstracts of all unique reports were screened, 35 reports underwent full-text screening. Finally, 6 reports qualified for inclusion in this secondary systematic review (Fig. S2).

### HCV antibody prevalence overview

We present here a synthesis of HCV prevalence in each country of CA. The 208 HCV prevalence measures included 67 measures from Kazakhstan, 96 from Kyrgyzstan, 20 from Tajikistan, 23 from Uzbekistan, and 2 from mixed-country samples. No study was identified from Turkmenistan (Fig. S8A).

#### Overall

In CA, HCV prevalence ranged from 0.5–13.1% among the general population, with a median of 2.0%. This included blood donors (number of studies (n) = 9), with HCV prevalence ranging from 0.9–7.3%, with a median of 1.5%; 0.5–6.0% among pregnant women (n = 9), with a median of 1.5%; and 0.7–13.1 among other general populations (n = 19), with a median of 2.0% (Table 1).

**Table 1:** Studies reporting hepatitis C virus (HCV) prevalence among the general population in Central Asia (CA).

Author, year (citation)	Year(s) of data collection	Country of survey	Study site	Study design	Study sampling	Population	Sample size	HCV prevalence (%)^a^
Skorikova, 2015^78^	2012	Kazakhstan	Blood transfusion center	CS	Conv	Blood donors	28,248	0.90
Nurgalieva, 2007^45^	NS	Kazakhstan	Community	CS	Conv	General population	150	2.0
El-Bassel, 2011^79^	2008	Kazakhstan	Community	CS	SRS	General population (female)	213	3.0
El-Bassel, 2011^79^	2008	Kazakhstan	Community	CS	SRS	General population (male)	209	0.0
Dzhumagalieva, 2015^80^	NS	Kazakhstan	Community	NS	NS	Pregnant women	300^ǂ^	5.1
Khasenova, 2007^81^	2006	Kazakhstan	National	CS	Conv	Pregnant women	6,405	1.0
Blood-center, 2015^82^	2015	Kazakhstan	Blood bank	CS	Conv	Blood donors	285,484	0.86
Tashtemirov, 2016^83^	2016	Kazakhstan	Blood bank	CS	Conv	Blood donors	59,323	0.85
Mamaev, 2006^84^	2005	Kyrgyzstan	Community	CS	Conv	Pregnant women	898	1.6
Djumagulova, 2016^85^	2011	Kyrgyzstan	Community	CS	Conv	Blood donors	37,771	2.6
Djumagulova, 2016^85^	2012	Kyrgyzstan	Community	CS	Conv	Blood donors	36,463	2.5
Djumagulova, 2016^85^	2013	Kyrgyzstan	Community	CS	Conv	Blood donors	37,463	2.5
Djumagulova, 2016^85^	2014	Kyrgyzstan	Community	CS	Conv	Blood donors	41,156	1.8
Djumagulova, 2016^85^	2015	Kyrgyzstan	Community	CS	Conv	Blood donors	42,038	1.9
Djumagulova, 2016^85^	2004	Kyrgyzstan	National	CS	Conv	General population	300^ǂ^	2.0
Djumagulova, 2016^85^	2005	Kyrgyzstan	National	CS	Conv	General population	300^ǂ^	1.0
Djumagulova, 2016^85^	2006	Kyrgyzstan	National	CS	Conv	General population	300^ǂ^	5.0
Djumagulova, 2016^85^	2007	Kyrgyzstan	National	CS	Conv	General population	300^ǂ^	5.0
Djumagulova, 2016^85^	2008	Kyrgyzstan	National	CS	Conv	General population	300^ǂ^	2.0
Djumagulova, 2016^85^	2009	Kyrgyzstan	National	CS	Conv	General population	300^ǂ^	2.0
Djumagulova, 2016^85^	2010	Kyrgyzstan	National	CS	Conv	General population	300^ǂ^	5.0
Djumagulova, 2016^85^	2011	Kyrgyzstan	National	CS	Conv	General population	300^ǂ^	0.80
Djumagulova, 2016^85^	2012	Kyrgyzstan	National	CS	Conv	General population	300^ǂ^	4.0
Djumagulova, 2016^85^	2013	Kyrgyzstan	National	CS	Conv	General population	300^ǂ^	2.0
Djumagulova, 2016^85^	2014	Kyrgyzstan	National	CS	Conv	General population	300^ǂ^	5.0
Djumagulova, 2016^85^	2013	Kyrgyzstan	National	CS	Conv	Pregnant women	300^ǂ^	1.0
Djumagulova, 2016^85^	2014	Kyrgyzstan	National	CS	Conv	Pregnant women	300^ǂ^	1.4
Djumagulova, 2016^85^	2015	Kyrgyzstan	National	CS	Conv	Pregnant women	300^ǂ^	1.6
Djumagulova, 2016^85^	2013	Kyrgyzstan	National	CS	Conv	Army recruits	300^ǂ^	1.0
Djumagulova, 2016^85^	2014	Kyrgyzstan	National	CS	Conv	Army recruits	300^ǂ^	1.0
Bakhovadinov, 2016^86^	2016	Kyrgyzstan	Blood bank	CS	Conv	Blood donors	46,780	1.8
Bahovadinov, 2010^87^	2007–2009	Tajikistan	Community	CS	Conv	Blood donors	66,333	2.9
Asimov, 2015^41^	2006–2010	Tajikistan	Community	CS	SRS	Pregnant women	315	6.0
Asimov, 2015^41^	2006–2010	Tajikistan	Community	CS	SRS	Paid blood donors	68	7.3
Abdurashit, 2008^88^	2005–2007	Tajikistan	National	CS	Conv	Pregnant women	1,554	0.50
Aklsalikh, 2017^40^	NS	Tajikistan	Community	CS	Conv	Labor workers	415	4.8
Aklsalikh, 2017^40^	NS	Uzbekistan	Community	CS	Conv	Labor workers	464	4.5
Kurbanov, 2003^43^	2001	Uzbekistan	Clinical	CS	Conv	Blood donors, pregnant women	341	6.5
Ruzibakiev, 2001^89^	1999–2000	Uzbekistan	Community	CS	SRS	General population	929	11.3
Ruzibakiev, 2001^89^	1999–2000	Uzbekistan	Community	CS	SRS	Paid blood donors	346	6.4
Berger, 2015^90^	1999–2000	Uzbekistan	Community	NS	NS	General population	300^ǂ^	13.1
Glikberg, 1997	1995–1997	Israel^¥^	Community	CS	Conv	General population (Bukharian Jews)	102	26.5

Abbreviations: Conv, convenience; CS, cross-sectional; NS, not specified; SRS, simple random sampling.

^a^Prevalence figures are as reported in the original reports, but rounded to one decimal place, provided the prevalence figure was over 1%.

^**ǂ**^Study did not report sample size. The included sample size was imputed based on the median sample size of all studies that reported a sample size.

^¥^Study performed on immigrants from Central Asia.

HCV prevalence ranged from 0.0–50.0% among populations at intermediate risk, with a median of 13.2%. These included prisoners, with HCV prevalence ranging from 7.0–50.0%, with a median of 32.0%; 0.0–28.0% among sex workers (male, female, unspecified), with a median of 11.0%; and 2.0–6.2% among HCW, with a median of 2.7% (Table S2).

HCV prevalence ranged from 4.0–40.3% among non-specific clinical populations, with a median of 8.5%. These included hospitalized populations with HCV prevalence ranging from 5.9–33.3%, with a median of 8.0%; and HIV patients with HCV prevalence ranging from 10.5–40.3%, with a median of 21.8% (Table 2).

**Table 2:** Studies reporting hepatitis C virus (HCV) prevalence among clinical populations in Central Asia (CA).

Author, year (citation)	Year(s) of data collection	Country of survey	Study site	Study design	Study sampling	Population	Sample size	HCV prevalence (%)^a^
**Non-specific clinical populations**								
Begaidarova, 2016^38^	NS	Kazakhstan	Clinical	CS	Conv	HIV patients	181	40.3
Djumagulova, 2016^85^	2004	Kyrgyzstan	National	CS	Conv	Clinical populations	300^ǂ^	9.0
Djumagulova, 2016^85^	2005	Kyrgyzstan	National	CS	Conv	Clinical populations	300^ǂ^	8.0
Djumagulova, 2016^85^	2006	Kyrgyzstan	National	CS	Conv	Clinical populations	300^ǂ^	8.0
Djumagulova, 2016^85^	2007	Kyrgyzstan	National	CS	Conv	Clinical populations	300^ǂ^	8.0
Djumagulova, 2016^85^	2008	Kyrgyzstan	National	CS	Conv	Clinical populations	300^ǂ^	8.0
Djumagulova, 2016^85^	2009	Kyrgyzstan	National	CS	Conv	Clinical populations	300^ǂ^	7.0
Djumagulova, 2016^85^	2010	Kyrgyzstan	National	CS	Conv	Clinical populations	300^ǂ^	8.0
Djumagulova, 2016^85^	2011	Kyrgyzstan	National	CS	Conv	Clinical populations	300^ǂ^	8.0
Djumagulova, 2016^85^	2012	Kyrgyzstan	National	CS	Conv	Clinical populations	300^ǂ^	7.0
Djumagulova, 2016^85^	2013	Kyrgyzstan	National	CS	Conv	Clinical populations	300^ǂ^	5.9
Djumagulova, 2016^85^	2014	Kyrgyzstan	National	CS	Conv	Clinical populations	300^ǂ^	19.1
Djumagulova, 2016^85^	2015	Kyrgyzstan	National	CS	Conv	Clinical populations	300^ǂ^	33.3
Djumagulova, 2016^85^	2014	Kyrgyzstan	National	CS	Conv	HIV patients	5,505	10.5
Djumagulova, 2016^85^	2015	Kyrgyzstan	National	CS	Conv	HIV patients	6,110	11.5
Asimov, 2015^41^	2006–2010	Tajikistan	Community	CS	SRS	HIV patients	109	32.1
Kurbanov, 2003^43^	2001	Uzbekistan	Clinical	CS	Conv	Hematological disease patients	186	26.9
Ruzibakiev, 2001^89^	1999–2000	Uzbekistan	Community	CS	SRS	Hematological disease patients	72	29.2
Ruzibakiev, 2001^89^	1999–2000	Uzbekistan	Community	CS	SRS	Renal disease patients	85	16.5
Djumagulova, 2016^85^	2013	Kyrgyzstan	National	CS	Conv	Recipients (blood, tissue, organs, sperm)	300^ǂ^	4.0
Djumagulova, 2016^85^	2014	Kyrgyzstan	National	CS	Conv	Recipients (blood, tissue, organs, sperm)	300^ǂ^	4.0
**Populations with liver-related conditions**								
Kurbanov, 2003^43^	2001	Uzbekistan	Clinical	CS	Conv	Acute hepatitis patients	240	20.0
Kurbanov, 2003^43^	2001	Uzbekistan	Clinical	CS	Conv	Chronic liver disease patients	234	41.9
Ruzibakiev, 2001^89^	1999–2000	Uzbekistan	Community	CS	SRS	Acute hepatitis patients	96	16.6
Ruzibakiev, 2001^89^	1999–2000	Uzbekistan	Community	CS	SRS	Chronic liver disease patients	164	26.8
Mirojov, 2013^39^	NS	Tajikistan	Community	CS	NS	Liver cirrhosis patients	1,374	36.0
Ni, 2012^91^	2002–2010	China^¥^	Community	CS	Conv	Primary liver cancer patients	335	40.4
Khan, 2008^42^	2006	Tajikistan	Clinical	CS	Conv	Patients with chronic liver disease	124	46.0
Nersesov, 2017^92^	2017	Kazakhstan	Clinical	CS	Conv	Hepatocellular carcinoma patients	1,357	23.8
Baimakhanov, 2017^86^	2017	Kazakhstan	Clinical	CS	Conv	Liver transplant patients	64	26.6

Abbreviations: Conv, convenience; CS, cross-sectional; NS, not specified; SRS, simple random sampling;

^a^Prevalence figures are as reported in the original reports, but rounded to one decimal place, provided the prevalence figure was over 1%.

^**ǂ**^Study did not report sample size. The included sample size was imputed based on the median sample size of all studies that reported a sample size.

^¥^Study performed on immigrants from Central Asia.

HCV prevalence ranged from 16.6–46.0% among populations with liver-related conditions, with a median of 26.8; and 17.0–90.2% among PWID, with a median of 51.0% (Table 3).

**Table 3:** Studies reporting hepatitis C virus (HCV) prevalence among people who inject drugs (PWID) in Central Asia (CA).

Author, year (citation)	Year(s) of data collection	Country of survey	Study site	Study design	Study sampling	Population	Sample size	HCV prevalence (%)^a^
Deryabina, 2015^93^	NS	Kazakhstan	Community	CS	Conv	PWID	300^ǂ^	63.0
Zhussupov, 2007^94^	2002	Kazakhstan	Community, NSP clinics	CS	Conv, SBS	PWID	1,426	79.8
Gilbert, 2010^95^	2005–2006	Kazakhstan	NSP clinic	CS	Conv	PWID	80	58.9
El-Bassel, 2014^96^	2009–2012	Kazakhstan	Community, NSP and HIV clinics	RCT^b^	Conv, SBS	PWID and non-injecting or injecting partners	600	77.0
El-Bassel, 2014^97^	2009–2012	Kazakhstan	Community, NSP and HIV clinics	RCT^b^	Conv, SBS	PWID (females)	194	89.8
El-Bassel, 2013^98^	2009–2012	Kazakhstan	Community, NSP and HIV clinics	RCT^b^	Conv, SBS	PWID	580	90.2
Zabransky, 2014^99^	2003	Kazakhstan	Community	CS	Conv	PWID	300^ǂ^	57.2
Zabransky, 2014^99^	2004	Kazakhstan	Community	CS	Conv	PWID	300^ǂ^	57.2
Zabransky, 2014^99^	2005	Kazakhstan	Community	CS	Conv	PWID	300^ǂ^	63.1
Zabransky, 2014^99^	2006	Kazakhstan	Community	CS	Conv	PWID	300^ǂ^	52.6
Zabransky, 2014^99^	2007	Kazakhstan	Community	CS	Conv	PWID	300^ǂ^	47.6
Zabransky, 2014^99^	2008	Kazakhstan	Community	CS	Conv	PWID	300^ǂ^	64.1
Zabransky, 2014^99^	2009	Kazakhstan	Community	CS	Conv	PWID	300^ǂ^	60.3
Zabransky, 2014^99^	2010	Kazakhstan	Community	CS	Conv	PWID	300^ǂ^	58.7
Zabransky, 2014^99^	2011	Kazakhstan	Community	CS	Conv	PWID	300^ǂ^	61.2
Soliev, 2010^100^	2009	Kazakhstan	National	CS	Conv	PWID	4,860	60.0
Ganina, 2016^101^	2013	Kazakhstan	National	CS	Conv	PWID		60.3
Ganina, 2016^101^	2014	Kazakhstan	National	CS	Conv	PWID	4,414	70.7
Rosenkranz, 2016^102^	2016	Kazakhstan	Narcological Centers and Community	CS	Conv	PWID	600	43.3
Djumagulova, 2016^85^	2013	Kyrgyzstan	National	CS	Conv	PWID	300^ǂ^	31.9
Djumagulova, 2016^85^	2014	Kyrgyzstan	National	CS	Conv	PWID	300^ǂ^	40.4
Djumagulova, 2016^85^	2015	Kyrgyzstan	National	CS	Conv	PWID	300^ǂ^	35.2
Djumagulova, 2016^85^	2004	Kyrgyzstan	National	CS	Conv	PWID	300^ǂ^	56.0
Djumagulova, 2016^85^	2005	Kyrgyzstan	National	CS	Conv	PWID	300^ǂ^	40.0
Djumagulova, 2016^85^	2006	Kyrgyzstan	National	CS	Conv	PWID	300^ǂ^	45.0
Djumagulova, 2016^85^	2007	Kyrgyzstan	National	CS	Conv	PWID	300^ǂ^	52.0
Djumagulova, 2016^85^	2008	Kyrgyzstan	National	CS	Conv	PWID	300^ǂ^	44.0
Djumagulova, 2016^85^	2009	Kyrgyzstan	National	CS	Conv	PWID	300^ǂ^	31.0
Djumagulova, 2016^85^	2010	Kyrgyzstan	National	CS	Conv	PWID	300^ǂ^	17.0
Djumagulova, 2016^85^	2011	Kyrgyzstan	National	CS	Conv	PWID	300^ǂ^	34.0
Djumagulova, 2016^85^	2012	Kyrgyzstan	National	CS	Conv	PWID	300^ǂ^	53.0
Zabransky, 2014^99^	2005	Kyrgyzstan	Community	CS	Conv	PWID	300^ǂ^	50.6
Zabransky, 2014^99^	2006	Kyrgyzstan	Community	CS	Conv	PWID	300^ǂ^	48.4
Zabransky, 2014^99^	2007	Kyrgyzstan	Community	CS	Conv	PWID	300^ǂ^	51.3
Zabransky, 2014^99^	2008	Kyrgyzstan	Community	CS	Conv	PWID	300^ǂ^	47.5
Zabransky, 2014^99^	2009	Kyrgyzstan	Community	CS	Conv	PWID	300^ǂ^	53.7
Zabransky, 2014^99^	2010	Kyrgyzstan	Community	CS	Conv	PWID	300^ǂ^	50.4
Soliev, 2010^100^	2009	Kyrgyzstan	National	CS	Conv	PWID	900	54.0
Drew, 2005^103^	2004	Kyrgyzstan	NS	NS	NS	PWID	200	45.0
Drew, 2005^103^	2004	Kyrgyzstan	NS	NS	NS	PWID	265	60.0
Rosenkranz, 2016^102^	2016	Kyrgyzstan	Narcological Centers and Community	CS	Conv	PWID	900	21.2
Asimov, 2015^41^	2006–2010	Tajikistan	Community	CS	SRS	PWID	315	40.9
Beyrer, 2008^44^	2004	Tajikistan	Community, NSP clinic	CS	Conv, SBS	PWID	240	67.1
Zabransky, 2014^99^	2005	Tajikistan	Community	CS	Conv	PWID	300^ǂ^	43.1
Zabransky, 2014^99^	2006	Tajikistan	Community	CS	Conv	PWID	300^ǂ^	45.0
Zabransky, 2014^99^	2007	Tajikistan	Community	CS	Conv	PWID	300^ǂ^	31.1
Zabransky, 2014^99^	2008	Tajikistan	Community	CS	Conv	PWID	300^ǂ^	29.9
Zabransky, 2014^99^	2009	Tajikistan	Community	CS	Conv	PWID	300^ǂ^	32.6
Zabransky, 2014^99^	2010	Tajikistan	Community	CS	Conv	PWID	300^ǂ^	27.8
Zabransky, 2014^99^	2011	Tajikistan	Community	CS	Conv	PWID	300^ǂ^	24.9
Soliev, 2010^100^	2009	Tajikistan	National	CS	Conv	PWID	1,657	33.0
Kurbanov, 2003^104^	2001	Uzbekistan	Clinical	CS	Conv	PWID	60	51.7
Ruzibakiev, 2001^89^	1999–2000	Uzbekistan	Community	CS	SRS	PWID	51	62.7
Beyrer, 2008^44^	2004	Uzbekistan	Community, NSP clinic	CS	Conv, SBS	PWID	58	63.8
Zabransky, 2014^99^	2005	Uzbekistan	Community	CS	Conv	PWID	300^ǂ^	53.7
Zabransky, 2014^99^	2007	Uzbekistan	Community	CS	Conv	PWID	300^ǂ^	35.5
Zabransky, 2014^99^	2009	Uzbekistan	Community	CS	Conv	PWID	300^ǂ^	28.5
Zabransky, 2014^99^	2011	Uzbekistan	Community	CS	Conv	PWID	300^ǂ^	20.9
Inogamov, 2008^105^	2007	Uzbekistan	National	CS	Conv	PWID	3,743	36.0

Abbreviations: Conv, convenience; CS, cross-sectional; NS, not specified; SRS, simple random sampling; PWID, people who inject drugs; RCT, randomized controlled trial; SBS, snowball sampling; NSP, needle and syringe exchange program; HIV, human immunodeficiency virus.

^a^Prevalence figures are as reported in the original reports, but rounded to one decimal place, provided the prevalence figure was over 1%.

^b^In randomized controlled trials the extracted HCV prevalence measure was the cross-sectional baseline HCV prevalence measure.

^**ǂ**^Study did not report sample size. The included sample size was imputed based on the median sample size of all studies that reported a sample size.

#### Country-level

In Kazakhstan, HCV prevalence ranged from 0.7–5.1% among the general population, with a median of 0.9%; and 2.0–50.0% among populations at intermediate risk, with a median of 29.0%. Only one study was identified among non-specific clinical populations, with an HCV prevalence of 40.3% in HIV patients^38^. HCV prevalence ranged from 23.8–40.4% in populations with liver-related conditions, with a median of 26.6%; and 43.3–90.2% among PWID, with a median of 60.3%.

In Kyrgyzstan, HCV prevalence ranged from 0.8–5.0% among the general population, with a median of 2.0%; 0.0–35.0% among populations at intermediate risk, with a median of 7.0%; and 4.0–33.3% among non-specific clinical populations, with a median of 8.0%. No studies were identified among populations with liver-related conditions. HCV prevalence ranged from 17.0–60.4% among PWID, with a median of 46.4%.

In Tajikistan, HCV prevalence ranged from 0.5–7.3% among the general population, with a median of 3.9%. Only two studies were conducted among populations at intermediate risk^39^, with HCV prevalence of 4.2% among sex workers (male, female, unspecified)^40^, and 6.2% among HCW^41^. Only one study was conducted on non-specific clinical populations, with an HCV prevalence of 32.1% in HIV patients^41^. Only two studies were conducted on populations with liver-related conditions, reporting an HCV prevalence of 46.0%^42^ and 36.0%^39^. HCV prevalence ranged from 24.9–67.1% among PWID, with a median of 32.6%.

No studies were identified from Turkmenistan.

In Uzbekistan, HCV prevalence among the general population ranged from 6.4–13.1%, with a median of 6.5%; 9.2–18.8% among populations at intermediate risk, with a median of 11.9%; 16.5–29.2% among non-specific clinical populations, with a median of 26.9%; 16.6–41.9% among populations with liver-related conditions, with a median of 23.4%; and 20.9–63.8% among PWID, with a median of 51.7%.

### Pooled mean HCV prevalence estimates and estimated number of HCV infected persons

The national population-level HCV prevalence for each country, based on pooling the general population measures, were estimated at: 0.7% (95%CI: 0.7–0.8%) in Kazakhstan, 2.0% (95%CI: 1.7–2.4%) in Kyrgyzstan, 2.6% (95%CI: 1.7–3.6%) in Tajikistan, and 9.6% (95%CI: 5.8–14.2%) in Uzbekistan. For all countries combined, the pooled mean HCV prevalence was estimated at 2.2% (95%CI: 1.9–2.6%). Figure S8B maps the pooled mean HCV prevalence estimates for CA.

Across CA, the estimated pooled mean HCV prevalence was 14.6% (95%CI: 12.8–16.5%) among populations at intermediate risk; 13.5% (95%CI: 10.9–16.4%) among non-specific clinical populations; 31.6% (95%CI: 5.8–37.7%) among populations with liver-related conditions; and 51.3% (95%CI: 46.9–55.6%) among PWID. The results of pooling these populations for each country separately can be found in Table 4.

**Table 4:** Meta-analyses for hepatitis C virus (HCV) prevalence in Central Asia (CA) by risk population.

	Studies	Samples	Prevalence	Pooled HCV prevalence	Heterogeneity measures			Pooled chronic infection prevalence	Population size^46^	Estimated number of HCV antibody positive persons	Estimated number of HCV chronically-infected persons
	Total n	Total N	Range (%)^¥^	Mean (95% CI)	Q (p-value)ª	I² (confidence limits)^b^	Prediction interval (%)^c^	Mean (95% CI)			
**Kazakhstan**											
General population	14	665,859	0.0–5.1	0.7 (0.7–0.8)	75.8 (p < 0.01)	82.9% (72.25–89.3%)	0.5–1.0	0.5 (0.5–0.5)	18,403,860	128,827 (128,827–147,231)	87,087 (87,087–99,528)
Populations at intermediate risk	36	13,175	2.0–50.0	24.4 (19.3–29.9)	1767.3 (p = 0)	98.0% (97.7–98.3%)	1.7–61.5				
Non-specific clinical populations	—	—	—	—	—	—	—				
Populations with liver-related conditions	3	1,756	23.8–40.4	30.1 (18.6–43.0)	34.2 (p < 0.01)	94.1% (86.3–97.5%)	0.0–100				
People who inject drugs	20	20,549	43.3–90.6	66.7 (61.8–71.5)	894.1 (p < 0.01)	97.9% (97.4–98.3%)	42.6–87.0				
**Kyrgyzstan**											
General population	22	200,560	0.7–5.0	2.0 (1.7–2.4)	195.8 p < 0.01)	89.3% (85.1–92.3%)	1.1–3.2	1.4 (1.2–1.6)	6,132,932	122,659 (104,260–147,190)	82,917 (70,480–99,501)
Populations at intermediate risk	42	206,130	0.0–42.4	8.6 (7.3–10.0)	3560.1 (p = 0)	98.8% (98.7–99.0%)	2.1–18.6				
Non-specific clinical populations	16	15,815	4.0–33.3	9.3 (7.5–11.4)	188.5 (p < 0.01)	92.0% (88.7–94.4%)	2.9–18.8				
Populations with liver-related conditions	—	—	—	—	—	—	—				
People who inject drugs	22	7,715	17.0–60.4	43.4 (37.9–49.0)	512.4 (p < 0.01)	95.9% (94.8–96.8%)	18.2–70.6				
**Tajikistan**											
General population	6	115,465	0.5–7.4	2.6 (1.7–3.6)	219.6 (p < 0.01)	98.1% (97.1–98.8%)	0.4–6.4	1.8 (1.2–2.4)	9,107,211	236,787 (154,823–327,860)	160,068 (104,660–221,633)
Populations at intermediate risk	—	—	—	—	—	—	—				
Non-specific clinical populations	—	—	—	—	—	—	—				
Populations with liver-related conditions	3	1,498	36.0–47.5	40.6 (32.7–48.8)	4.9 (p = 0.09)	59.0% (0.0–88.3%)	0.0–100				
People who inject drugs	11	2,953	24.9–67.1	42.4 (33.6–51.4)	247.1 (p < 0.01)	96.0% (94.2–97.2%)	12.0–76.4				
**Uzbekistan**											
General population	6	2,411	4.5–29.0	9.6 (5.8–14.2)	50.8 (p < 0.01)	90.1% (82.1–94.5%)	0.3–28.1	6.5 (3.9–9.6)	32,364,996	3,107,040 (1,877,170–4,595,829)	2,100,359 (1,268,967–3,106,781)
Populations at intermediate risk	5	2,222	9.2–18.8	13.8 (11.1–16.9)	12.3 (p = 0.03)	59.3% (0.0–83.4%)	6.7–23.2				
Non-specific clinical populations	4	734	16.5–53.8	26.1 (15.8–37.9)	35.2 (p < 0.01)	82.8% (56.0–93.3%)	0.0–82.3				
Populations with liver-related conditions	4	382	16.6–41.9	29.8 (18.6–42.4)	17.4 (p < 0.01)	91.5% (81.3–96.1%)	0.0–84.9				
People who inject drugs	7	1,369	20.9–63.8	43.9 (31.8–56.4)	119.6 (p < 0.01)	95.0% (91.9–96.9%)	7.3–85.0				
**All countries**											
General population	49	984,397	0.0–29.0	2.2 (1.9–2.6)	3,707.0 (p = 0)	98.7% (98.6–98.8%)	0.5–4.6	1.5 (1.3–1.8)	66,008,999	3,595,313 (2,265,079–5,218,110)	2,430,431 (1,531,194–3,527,443)
Populations at intermediate risk	87	229,619	0.0–50.0	14.6 (12.8–16.5)	11,442.8 (p = 0)	99.2% (99.2–99.3%)	2.2–35.1				
Non-specific clinical populations	22	16,487	4.0–53.9	13.5 (10.9–16.4)	400.0 (p < 0.01)	94.8% (93.2–96.0%)	3.4–28.8				
Populations with liver-related conditions	10	3,988	16.7–47.5	31.6 (25.8–37.7)	114.8 (p < 0.01)	92.2% (87.7–95.0%)	12.7–54.3				
People who inject drugs	60	32,586	17.0–90.6	51.3 (46.9–55.6)	3561.5 (p = 0)	98.3% (98.2–98.5%)	19.1–82.8				

Abbreviations: CI, confidence interval

ªQ: Cochran Q statistic assesses if heterogeneity is present in HCV prevalence estimates.

^**b**^I²: Assesses the percentage of between-study variation that is due to true differences in HCV prevalence estimates across studies rather than chance.

^**c**^Prediction interval: Estimates the 95% interval in which the true HCV prevalence in a new HCV study will lie.

^¥^This range is for all studies included in the meta-analyses database and covers the range of HCV prevalence across not only main HCV prevalence measures, but also across all strata.

Forest plots for the meta-analyses can be found in the Supplementary Material (Figs S3–S7). In the majority of meta-analyses, statistically significant heterogeneity was observed (Cochrane’s Q statistic’s p-value was always <0.0001; Table 4). Most of the variation across studies was due to variation in effect size (HCV prevalence) rather than chance (I^2^ > 59.0%). The prediction intervals ranged from narrow to wide for the different meta-analyses. Collectively, the heterogeneity measures indicated high heterogeneity in HCV prevalence in each country and risk population category.

Too few studies reported HCV RNA viremic rate in the general population to warrant calculation of the pooled mean viremic rate for CA. Accordingly, the pooled mean viremic rate of 67.6% for MENA was used in calculating chronic-infection prevalence and the number of chronically-infected persons. This choice is justified by the fact that this measure is a biological measure that (in principle) should be largely independent of the region^31^, and given that CA and MENA countries are both developing countries. The highest number of chronically-infected persons was found in Uzbekistan at 2.1 million, followed by Tajikistan at 160,068, Kazakhstan at 87,087, and Kyrgyzstan at 82,917.

In sensitivity analyses, the GLMM meta-analyses confirmed similar results for all risk populations (Table S3). Also in sensitivity analyses, after blood donor data were excluded, population-level HCV prevalence was overall similar across countries, and in CA as a whole (Table S4).

### Meta-regressions and sources of heterogeneity

The results of the meta-regression for the general population is presented in Table 5. In the univariable meta-regression analyses, country, study site, sample size, and year of data collection were significant predictors (p-value < 0.1), and therefore were included in the final multivariable analysis. Notably, sampling method (probability-based versus non-probability-based) had no effect on observed HCV prevalence.

**Table 5:** Univariable and multivariable meta-regression models for hepatitis C virus (HCV) prevalence among the general population in Central Asia (CA).

		Univariable analysis				Multivariable analysis^a^	
		Number of studies	*OR* (95% CI)	p-value	Variance explained adjusted R^2^ (%)	*AOR* (95% CI)	p-value
Country	Kazakhstan	14	1	—		1	—
	Kyrgyzstan	22	2.0 (1.2–3.3)	0.006		2.0 (1.1–3.4)	0.015
	Tajikistan	6	3.0 (1.5–6.1)	0.003		2.8 (1.4–5.6)	0.006
	Uzbekistan	6	11.2 (5.6–22.7)	0.000	49.9	10.0 (4.6–21.7)	0.000
Low risk subpopulation	Blood donors	18	1	—		—	—
	General populations	22	1.6 (0.9–3.1)	0.134		—	—
	Pregnant women	8	1.0 (0.4–2.4)	0.942	1.5	—	—
Study site	Community	23	1	—		1	—
	Blood bank	8	0.6 (0.3–1.4)	0.229		0.9 (0.5–1.7)	0.745
	Antenatal clinics	17	0.5 (0.3–1.0)	0.061	4.2	0.8 (0.5–1.4)	0.438
Sample size	<100	3	1	—		1	—
	≥100	45	0.3 (0.1–0.9)	0.028	8.2	0.4 (0.1–1.0)	0.043
Sampling method	Probability-based	6	1			1	—
	Non-probability-based	40	0.5 (0.2–1.2)	0.126	3.1		
Year of data collection		48	0.9 (0.9–1.0)	0.026	8.4	1.0 (1.0–1.1)	0.654
Year of publication		48	1.0 (0.9–1.0)	0.149	2.4	—	—

Abbreviations: OR, odds ratio; AOR, adjusted odds ratio; CI, confidence interval.

^a^The adjusted R-squared for the full model was 51.4%.

Study site and year of data collection lost significance (p-value > 0.05) in the multivariable analysis—only country and sample size remained statistically significant. Relative to Kazakhstan, the prevalence in Kyrgyzstan, Tajikistan, and Uzbekistan was higher with an adjusted odds ratio (AOR) of 2.0 (95%CI: 1.1–3.4), 2.8 (95%CI: 1.4–5.6), and 10.0 (95%CI: 4.6–21.7), respectively. Sample size (>100) was associated with lower HCV prevalence, with an AOR of 0.4 (95%CI: 0.1–1.0). Notably, the AOR for year of data collection was 1.0 (95%CI: 1.0–1.1)—there was thus no evidence for declines in HCV prevalence with time. The model explained 51.4% of the variability in HCV prevalence.

### HCV RNA prevalence

Our search identified only four HCV RNA measures, all of which were reported among HCV antibody-positive individuals: 39.2% in a study on a general population^43^, 100% in a study on HIV patients^38^, 100% in a study on chronic hepatitis patients^42^, and 70.5% in a study on liver cirrhosis patients^42^.

### HCV genotypes

HCV genotype information was available in six studies with a total of 382 HCV RNA positive individuals (Table S5). Only 0.5% of individuals were infected with multiple genotypes, while the remaining majority were infected with a single genotype. No genotype information was available for Kyrgyzstan and Turkmenistan.

The highest proportions of infections for each HCV genotype in CA as a whole were for genotype 1 at 52.6% and genotype 3 at 38.0%, followed by genotype 2 at 9.4%. Genotypes 4, 5, 6, and 7 were not identified. Genotype diversity tended towards being low, but varied across CA, with the highest diversity observed in Kazakhstan (H = 1.04 out of 1.95; 53.7%), followed by Uzbekistan (H = 0.85 out of 1.95; 43.6%), and Tajikistan (H = 0.54 out of 1.95; 27.5%). Collectively in CA, genotype diversity was rather low (H = 0.93 out of 1.95; 47.7%).

### HCV risk factors

Only two studies reported statistically-significant risk factors for HCV infection after controlling for confounders. In Tajikistan, among PWID, daily injection, history of incarceration, and living/working outside of Tajikistan in the past 10 years, were associated with HCV infection^44^. In Kazakhstan, among a general population, tattooing and (unexpectedly) towel sharing were reported as associated with HCV infection^45^.

### Quality assessment

Table S6 summarizes the results of the ROB assessment performed on HCV prevalence measures. The majority of measures were of high precision (94.7%), with a sample size ≥100. Most measures were of low risk of bias in the HCV ascertainment domain, with 99% being based on biological assays, and 1% being based on self-reporting. Though most of the studies reported the name of the biological assay used to assess HCV antibody prevalence, the majority of studies (90%) did not explicitly report the generation of the assay. Among studies reporting the generation of the used assay, all used the more sensitive and specific 3^rd^ generation enzyme-linked immunosorbent assays (ELISA) tests. The majority of studies employed non-probability-based sampling, and were characterized by a high response rate.

To summarize, 100% of studies had low ROB based on at least one ROB domain, 65.0% of studies had low ROB based on at least two ROB domains, and 13.4% of studies had low ROB based on all three ROB domains. No study had high ROB based on two or three ROB domains. In all, the quality assessment indicates reasonable though not optimal study quality.

## Discussion

We presented, to our knowledge, the first systematic review and synthesis of HCV epidemiology in CA, a region perceived to be heavily affected by this infection^9,10^. Our results indicated that HCV antibody prevalence varies across countries of CA, ranging from 0.7% in Kazakhstan to 9.6% in Uzbekistan (Table 4 and Fig. S8). Accordingly, HCV prevalence in Uzbekistan is considerably higher than global levels, and one of the highest worldwide^9,10^. This finding is of concern considering that Uzbekistan is also the most populous country in CA, with 32 million inhabitants^46^, and a country struggling with a weakened healthcare system since the collapse of the Soviet Union^6^. With an estimated 2.1 million chronically-infected persons, >80% of all chronically-infected persons in CA reside in Uzbekistan. Notably, Uzbekistan has also the highest rate of HIV among all countries in this region^47^, and a main mode of transmission appears to be injecting drug use, a shared mode of transmission with HCV.

Remarkably, HCV prevalence does not appear to be decreasing with time in CA (Table 5), contrary to global trend^48,49^. This may in part be reflective of the majority of studies from this region being reported more recently, with approximately 85% of all studies included in this review being from the last decade.

High HCV antibody prevalence was observed across all risk populations (Tables 1–3 and S2), and more so for PWID, HIV patients, and prisoners, suggesting a major role for injecting drug use in infection transmission. HCV antibody prevalence was also high in populations with liver-related conditions, suggesting a major role that HCV plays in liver disease burden in CA.

Strikingly, no studies were identified among high risk clinical populations such as haemodialysis, haemophilia, and thalassemia patients—the role of healthcare in transmission remains uncertain. However, the relatively high HCV antibody prevalence in non-specific clinical populations (Table 2), and HCV epidemiology in other soviet-era-related countries^9,10,49,50^, suggest that healthcare could be a major mode of exposure, at least in earlier decades.

Subregional disparities in quality of healthcare services may have also contributed to the heterogeneity in HCV prevalence across CA^50^. For example, in Uzbekistan, it appears (anecdotally) that there is an excessive practice of medical and non-medical invasive procedures, such as blood transfusions and bloodletting, in addition to poor infection control^51^, inadequate blood screening^43,51,52^, and use of unsafe medical injections^50,51^, all of which are probable causes for the high HCV prevalence in this country, as has been observed in other developing countries^53–55^. Furthermore, the challenging political climate in Uzbekistan has prevented the introduction of up-to-date healthcare guidelines and effective approaches to reduce HCV transmission in healthcare settings^51,52^.

While no genotype information was available for Kyrgyzstan and Turkmenistan, pooled analysis on data from Kazakhstan, Tajikistan, and Uzbekistan suggest that HCV genotype 1 (53% of infections) and genotype 3 (38%) are the major circulating strains, but with strong presence of genotype 2 (9%; Table S5). While genotype 1 is common globally^10,56^, its major presence may reflect healthcare-related exposures, given the frequency of identifying this genotype in clinical populations in CA^42,43,57,58^, as well as the global association between this genotype and healthcare exposures^56^. The major presence of genotype 3 may be due to injecting drug use being a major driver of incidence, given the global association between this genotype and injecting drug use^56^, or may just reflect a sub-regional pattern—genotype 3 is the main circulating strain in each of Afghanistan^13,59^ and Pakistan^59^, both of which are neighbouring countries of CA.

The pooled mean HCV prevalence in PWID indicated that over half of this population is already exposed to HCV (Table 4), similar to global trends^60–62^. Notably, CA is geographically located along drug trafficking routes originating from Afghanistan^7,63^, and is believed to have one of the highest rates of injecting drug use in the world^64^. These factors further corroborate a major role for injecting drug use in transmission. Furthermore, with the high HCV prevalence found in prisoners (Table S2), incarceration could be influential in HCV transmission dynamics, just as in other regions^62,65^. The high prevalence observed among sex workers (male, female, unspecified; Table S2) may suggest also high rates of injecting drug use in these populations, as supported by HIV biobehavioral surveillance data—sexual and injecting networks could be overlapping hotspots of both HCV and HIV transmission^7,64^.

Despite progress in characterizing HCV epidemiology in CA, our study highlights key challenges and limitations to establishing a satisfactory understanding. Evidence varied by country, with no data identified from Turkmenistan (Fig. S8A). No data were available for high risk clinical populations, though healthcare could be a major mode of exposure, as it is in other soviet-era-related countries^9,10,50^, and in countries with similar stage of development, e.g. in MENA^11–19,66^. No data was identified for community-related exposures, e.g. informal healthcare, but such exposures could play a role as seen in other regions^67^. There was an insufficient number of studies reporting HCV RNA prevalence in CA, a measure that informs assessment of chronic-infection prevalence, as antibody prevalence reflects both current infection as well as past infection (that is persons who spontaneously cleared the infection or were treated)^68^.

Most available studies were descriptive—few had analytic epidemiologic designs where risk factors and modes of exposure could be ascertained. Most studies employed non-probability-based sampling, however, results of the meta-regressions indicated this had no effect on HCV prevalence in the general population, and therefore may not have limited the representativeness of reviewed data in our study. There was high heterogeneity in HCV prevalence measures (Table 4), but most heterogeneity (for the general population) was subsequently explained—differences by country were the main driver of prevalence variation (Table 5). A small-study effect was observed, with studies with a smaller sample size reporting higher HCV prevalence (Table 5), thereby potentially limiting the representativeness of reviewed data. HCV genotype data was relatively sparse, with no studies identified from Kyrgyzstan and Turkmenistan.

In spite of these limitations, a key strength of our study is that we identified a substantial number of studies, including a volume of unpublished data, in a significantly affected, but poorly understood region, thereby facilitating a synthesis of evidence and identification of knowledge gaps. A priority in addressing these gaps is to carry out nationally-representative probability-based and population-based surveys in each of these countries. Such surveys can yield a precise estimate of HCV prevalence, delineation of spatial variability in infection exposure, identification of modes of transmission, and assessment of HCV knowledge and attitudes, as has been done in recent years in other countries, e.g. in Egypt^15,69–74^ and Pakistan^11,75–77^.

## Conclusion

In context of inadequate and underfunded healthcare systems^8,52^, CA is one of the most affected regions by HCV infection. Uzbekistan, in particular, appears to be enduring one of the highest prevalence levels worldwide. HCV transmission appears to be driven by injecting drug use and healthcare exposures, with no evidence for declines in prevalence in recent years. Genotypes 1 and 3 are the most frequently-circulating strains, with some presence for genotype 2.

Our findings inform HCV response for public health planning, health service provision, development of HCV policy guidelines, and implementation of HCV programming to reduce transmission and associated disease burden. Achieving HCV elimination in CA by 2030 can only be accomplished by aggressive action and commitment, given the extent of challenges. There is an urgent need for expansion of affordable HCV testing and treatment for key populations, and targeted control based on settings of exposure. In context of this region being heavily affected by injecting drug use and the global opioid epidemic, harm reduction services must incorporate HCV services and be accessible to all PWID, by being expanded to all relevant settings, such as prisons. Nationally-representative probability-based population-based surveys must be conducted to precisely delineate HCV epidemiology in these countries and address the knowledge gaps, as identified in this study. Improving infection control in healthcare facilities is also warranted, such as through updating (otherwise outdated) clinical guidelines for healthcare workers^52^, and adopting safety-engineered syringes as recommended by WHO^28,29^.

## Supplementary information

Supplementary Material
