# Supplementary material for: The epidemiology of hepatitis C virus in Central Asia: Systematic review, meta-analyses, and meta-regression analyses
Source: Sci Rep. 2019 Feb 14;9:2090. doi: 10.1038/s41598-019-38853-8 (PMC6376025; doi:10.1038/s41598-019-38853-8)
Supplement: Supplementary file 1 — Supplementary Material [file 41598_2019_38853_MOESM1_ESM.docx]

**SUPPLEMENTARY MATERIAL**

**The epidemiology of hepatitis C virus in Central Asia: Systematic review, meta-analyses, and meta-regression analyses**

Welathanthrige S. P. Botheju ^aǂ^, Fawzi Zghyer ^aǂ^, Sarwat Mahmud ^bǂ^, Assel Terlikbayeva ^c^, Nabila El-Bassel ^d^, Laith J. Abu-Raddad ^b,e,f*^

^a^ Weill Cornell Medicine - Qatar, Cornell University, Qatar Foundation - Education City, Doha, Qatar

^b^ Infectious Disease Epidemiology Group, Weill Cornell Medicine - Qatar, Cornell University, Qatar Foundation - Education City, Doha, Qatar

^c^ Global Health Research Center of Central Asia in Kazakhstan, Almaty, Kazakhstan

^d^ Social Intervention Group, Columbia University School of Social Work, New York, New York, USA

^e^ Department of Healthcare Policy and Research, Weill Cornell Medicine, Cornell University, New York, New York, USA

^f^ College of Health and Life Sciences, Hamad bin Khalifa University, Doha, Qatar

ǂ Equal contributors

**Table S1.** Preferred Reporting Items for Systematic Reviews and Meta-analyses (PRISMA) checklist.

| **Section/topic** | **#** | **Checklist item** | **Reported in main text on** |
| --- | --- | --- | --- |
| **TITLE** | | |  |
| Title | 1 | Identify the report as a systematic review, meta-analysis, or both. | p. 1 |
| **ABSTRACT** | | |  |
| Structured summary | 2 | Provide a structured summary including, as applicable: background; objectives; data sources; study eligibility criteria, participants, and interventions; study appraisal and synthesis methods; results; limitations; conclusions and implications of key findings; systematic review registration number. | p. 2 |
| **INTRODUCTION** | | |  |
| Rationale | 3 | Describe the rationale for the review in the context of what is already known. | p. 3 |
| Objectives | 4 | Provide an explicit statement of questions being addressed with reference to participants, interventions, comparisons, outcomes, and study design (PICOS). | p. 3 |
| **METHODS** | | |  |
| Protocol and registration | 5 | Indicate if a review protocol exists, if and where it can be accessed (e.g., Web address), and, if available, provide registration information including registration number. | p. 4 |
| Eligibility criteria | 6 | Specify study characteristics (e.g., PICOS, length of follow-up) and report characteristics (e.g., years considered, language, publication status) used as criteria for eligibility, giving rationale. | p. 5 |
| Information sources | 7 | Describe all information sources (e.g., databases with dates of coverage, contact with study authors to identify additional studies) in the search and date last searched. | p. 4 |
| Search | 8 | Present full electronic search strategy for at least one database, including any limits used, such that it could be repeated. | Figure S1 |
| Study selection | 9 | State the process for selecting studies (i.e., screening, eligibility, included in systematic review, and, if applicable, included in the meta-analysis). | p. 4 |
| Data collection process | 10 | Describe method of data extraction from reports (e.g., piloted forms, independently, in duplicate) and any processes for obtaining and confirming data from investigators. | p. 5-6 |
| Data items | 11 | List and define all variables for which data were sought (e.g., PICOS, funding sources) and any assumptions and simplifications made. | p. 5 |
| Risk of bias in individual studies | 12 | Describe methods used for assessing risk of bias of individual studies (including specification of whether this was done at the study or outcome level), and how this information is to be used in any data synthesis. | p. 8-9 |
| Summary measures | 13 | State the principal summary measures (e.g., risk ratio, difference in means). | p. 5 |
| Synthesis of results | 14 | Describe the methods of handling data and combining results of studies, if done, including measures of consistency (e.g., I^2^) for each meta-analysis. | p. 7 |
| Risk of bias across studies | 15 | Specify any assessment of risk of bias that may affect the cumulative evidence (e.g., publication bias, selective reporting within studies). | p. 8 |
| Additional analyses | 16 | Describe methods of additional analyses (e.g., sensitivity or subgroup analyses, meta-regression), if done, indicating which were pre-specified. | p. 7 |
| **RESULTS** | | |  |
| Study selection | 17 | Give numbers of studies screened, assessed for eligibility, and included in the review, with reasons for exclusions at each stage, ideally with a flow diagram. | p. 9, Figure 2 |
| Study characteristics | 18 | For each study, present characteristics for which data were extracted (e.g., study size, PICOS, follow-up period) and provide the citations. | p. 10-11, Table 1, 2, 3, S2 |
| Risk of bias within studies | 19 | Present data on risk of bias of each study and, if available, any outcome level assessment (see item 12). | p. 14-15, Table S3 |
| Results of individual studies | 20 | For all outcomes considered (benefits or harms), present, for each study: (a) simple summary data for each intervention group (b) effect estimates and confidence intervals, ideally with a forest plot. | p. 8-12, Figure S2-S6 |
| Synthesis of results | 21 | Present results of each meta-analysis done, including confidence intervals and measures of consistency. | p. 37 Table 4 |
| Risk of bias across studies | 22 | Present results of any assessment of risk of bias across studies (see Item 15). | p. 13, 39, Table 5 |
| Additional analysis | 23 | Give results of additional analyses, if done (e.g., sensitivity or subgroup analyses, meta-regression [see Item 16]). | p. 12-14 |
| **DISCUSSION** | | |  |
| Summary of evidence | 24 | Summarize the main findings including the strength of evidence for each main outcome; consider their relevance to key groups (e.g., healthcare providers, users, and policy makers). | p. 15-18 |
| Limitations | 25 | Discuss limitations at study and outcome level (e.g., risk of bias), and at review-level (e.g., incomplete retrieval of identified research, reporting bias). | p. 17-18 |
| Conclusions | 26 | Provide a general interpretation of the results in the context of other evidence, and implications for future research. | p. 19 |
| **FUNDING** | | |  |
| Funding | 27 | Describe sources of funding for the systematic review and other support (e.g., supply of data); role of funders for the systematic review. | p. 1 |

**Figure S1.** Search criteria for systematically reviewing hepatitis C virus (HCV) antibody incidence and antibody prevalence data in Central Asia (CA).

**1) Literature search on PubMed:**
((((((((((Hepatitis C[MeSH Terms]) OR Hepatitis C antibodies[MeSH Terms]) OR Hepatitis C antigens[MeSH Terms]) OR Hepacivirus[MeSH Terms]) OR Hepatitis C[Text Word]) OR Hepacivirus[Text Word]) OR Hepatitis C antibodies[Text Word]) OR Hepatitis C antigens[Text Word]) OR HCV[Text Word])) AND ((((((((Kazakhstan[MeSH Terms] or Kazakhstan*[Text Word] or Kazakh[Text Word]))) OR ((Kyrgyzstan*[Text Word] or Kyrgyzstan[MeSH Terms] or Kyrgyz[Text Word]))) OR ((Uzbekistan*[Text Word] or Uzbekistan[MeSH Terms] or Uzbek[Text Word]))) OR ((Tajikistan*[Text Word] or Tajikistan[MeSH Terms] or Tajik[Text Word]))) OR ((Turkmenistan*[Text Word] or Turkmenistan[MeSH Terms] or Turks[Text Word]))) OR ((Central asia[MeSH Terms] or Central asia*[Text Word])))

Searched: 09/04/18
**Results: 95 citations**

**2) Literature search on EMBASE:
(Ovid 1988 to present)**
(exp Kazakhstan/ or Kazakhstan*.mp. or exp Kyrgyzstan/ or Krygyzstan*.mp. or exp Uzbekistan/ or Uzbekistan*.mp. or exp Turkmenistan/ or Turkmenistan*.mp. or exp Tajikistan/ or Tajikistan*.mp. or exp Central Asian/ or Central Asian.mp.) and (exp Hepatitis C/ or hepatitis C.mp. or exp hepatitis C antibody/ or exp hepatitis C antigen/ or exp Hepatitis C virus/ or HCV.mp. or hepacivirus.mp)

Searched: 09/04/18
**Results: 129 citations**

**3) Literature search on Russian scientific database (eLibrary.ru)**(Hepatitis)

Searched: 09/04/18
**Results: 547 citations**

**Figure S2.** Flow chart of article selection for the systematic review of hepatitis C virus (HCV) genotypes, adapted from the PRISMA 2009 guidelines [1].

**
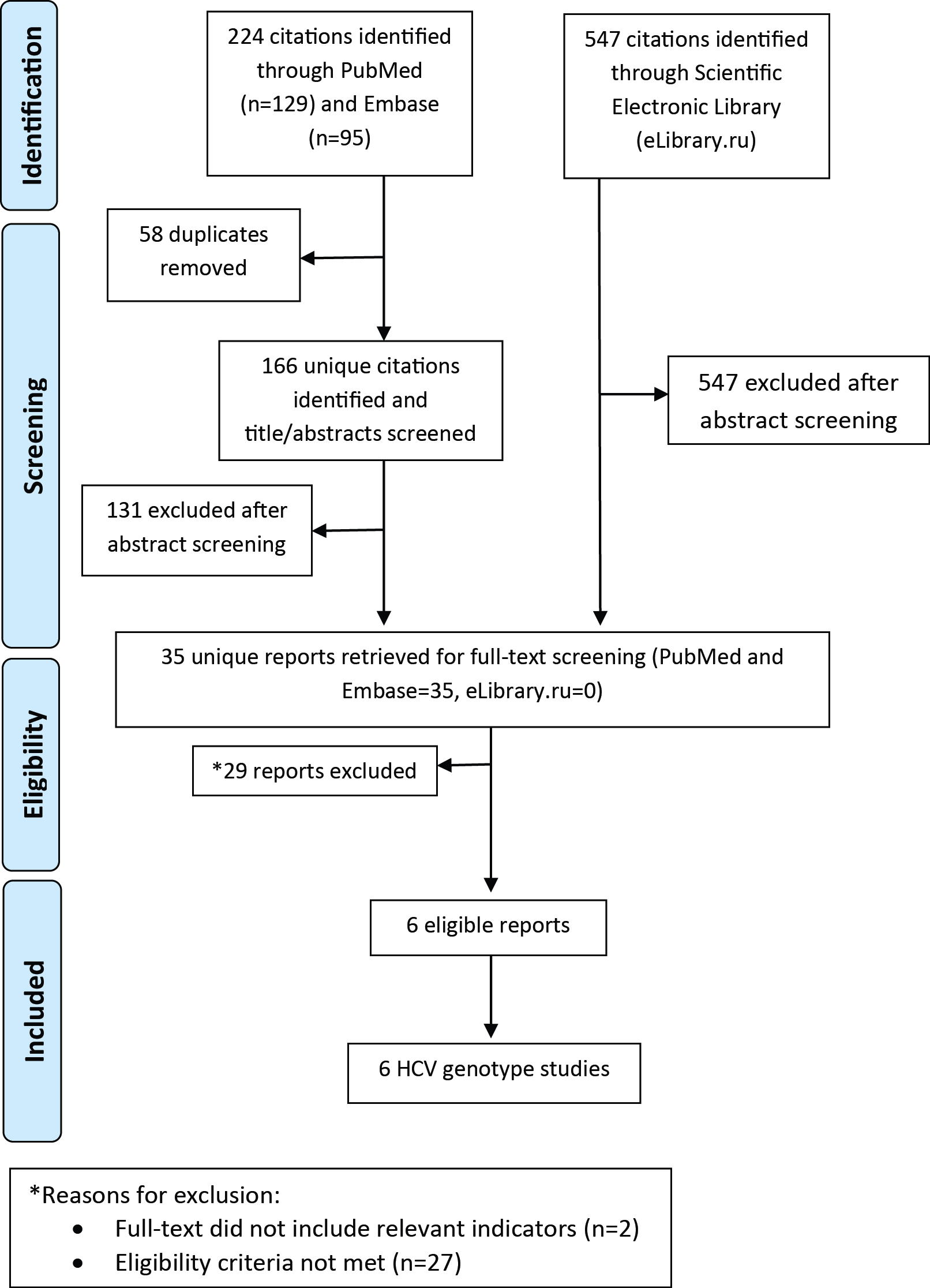
**

**Table S2.** Studies reporting hepatitis C virus (HCV) prevalence among intermediate risk populations in Central Asia (CA).

| Author, year (citation) | Year(s) of data collection | Country of survey | Study site | Study design | Study sampling | Population | Sample size | HCV prevalence (%)^a^ |
| --- | --- | --- | --- | --- | --- | --- | --- | --- |
| Ruzibakiev, 2001 [2] | 1999-2000 | Uzbekistan | Community | CS | SRS | Sex workers | 87 | 9.2 |
| Deryabina, 2015 [3] | NS | Kazakhstan | Community | CS | Conv | Sex partners of PWID | 300ǂ | 16.8 |
| El-Bassel, 2014 [4] | 2009-2012 | Kazakhstan | Community | RCT^b^ | Conv, SBS | Sex partners of PWID | 135 | 14.8 |
| El-Bassel, 2013 [5] | 2009-2012 | Kazakhstan | Community | RCT^b^ | Conv, SBS | Sex partners of PWID | 148 | 15.5 |
| Ismailova, 2010 [6] | 2009 | Kazakhstan | National | CS | Conv | MSM | 741 | 4.2 |
| Ongoeva, 2010 [7] | 2009 | Kazakhstan | National | CS | Conv | Sex workers | 2,249 | 11.0 |
| Sermoneta-Gertel, 2001 [8] | 1995-1997 | Israel¥ | Clinical | CS | Conv | HCW | 228 | 5.7 |
| Asimov, 2015 [9] | 2006-2010 | Tajikistan | Community | CS | SRS | HCW | 451 | 6.2 |
| Mehmondustovich, 2017 [10] | 2014 | Tajikistan | Community | CS | NS | Sex workers | 7,413 | 4.2 |
| Ismailova, 2010 [6] | 2009 | Kyrgyzstan | National | CS | Conv | MSM | 84 | 1.2 |
| Ongoeva, 2010 [7] | 2009 | Kyrgyzstan | National | NS | NS | Sex workers | 689 | 3.9 |
| Shapiro, 2003 [11] | 2003 | Kyrgyzstan | National | CS | Conv | Sex workers | 482 | 28.0 |
| Drew, 2005 [12] | 2004 | Kyrgyzstan | National | CS | Conv | Sex workers | 378 | 4.0 |
| Djumagulova, 2016 [13] | 2010 | Kyrgyzstan | National | CS | Conv | HCW | 41,825 | 2.2 |
| Djumagulova, 2016 [13] | 2011 | Kyrgyzstan | National | CS | Conv | HCW | 35,776 | 2.6 |
| Djumagulova, 2016 [13] | 2012 | Kyrgyzstan | National | CS | Conv | HCW | 36,955 | 3.9 |
| Djumagulova, 2016 [13] | 2013 | Kyrgyzstan | National | CS | Conv | HCW | 39,338 | 2.7 |
| Djumagulova, 2016 [13] | 2014 | Kyrgyzstan | National | CS | Conv | HCW | 40,885 | 3.5 |
| Djumagulova, 2016 [13] | 2015 | Kyrgyzstan | National | CS | Conv | HCW | 300ǂ | 2.4 |
| Botova, 2014 [14] | NS | Kazakhstan | Clinical | CC | Conv | HCW | 664 | 2.0 |
| Botova, 2014 [14] | NS | Kazakhstan | Clinical | CC | Conv | Inpatients with non-infectious diseases | 238 | 14.7 |
| Djumagulova, 2016 [13] | 2004 | Kyrgyzstan | National | CS | Conv | Prisoners | 300ǂ | 24.0 |
| Djumagulova, 2016 [13] | 2005 | Kyrgyzstan | National | CS | Conv | Prisoners | 300ǂ | 13.0 |
| Djumagulova, 2016 [13] | 2006 | Kyrgyzstan | National | CS | Conv | Prisoners | 300ǂ | 24.0 |
| Djumagulova, 2016 [13] | 2007 | Kyrgyzstan | National | CS | Conv | Prisoners | 300ǂ | 15.0 |
| Djumagulova, 2016 [13] | 2008 | Kyrgyzstan | National | CS | Conv | Prisoners | 300ǂ | 11.0 |
| Djumagulova, 2016 [13] | 2009 | Kyrgyzstan | National | CS | Conv | Prisoners | 300ǂ | 7.0 |
| Djumagulova, 2016 [13] | 2010 | Kyrgyzstan | National | CS | Conv | Prisoners | 300ǂ | 13.0 |
| Djumagulova, 2016 [13] | 2011 | Kyrgyzstan | National | CS | Conv | Prisoners | 300ǂ | 8.0 |
| Djumagulova, 2016 [13] | 2012 | Kyrgyzstan | National | CS | Conv | Prisoners | 300ǂ | 28.0 |
| Djumagulova, 2016 [13] | 2013 | Kyrgyzstan | National | CS | Conv | Prisoners | 300ǂ | 32.0 |
| Djumagulova, 2016 [13] | 2014 | Kyrgyzstan | National | CS | Conv | Prisoners | 300ǂ | 35.0 |
| Djumagulova, 2016 [13] | 2006 | Kyrgyzstan | National | CS | Conv | Contacts of PLHIV | 300ǂ | 22.0 |
| Djumagulova, 2016 [13] | 2007 | Kyrgyzstan | National | CS | Conv | Contacts of PLHIV | 300ǂ | 4.0 |
| Djumagulova, 2016 [13] | 2008 | Kyrgyzstan | National | CS | Conv | Contacts of PLHIV | 300ǂ | 3.0 |
| Djumagulova, 2016 [13] | 2009 | Kyrgyzstan | National | CS | Conv | Contacts of PLHIV | 300ǂ | 22.0 |
| Djumagulova, 2016 [13] | 2010 | Kyrgyzstan | National | CS | Conv | Contacts of PLHIV | 300ǂ | 11.0 |
| Djumagulova, 2016 [13] | 2011 | Kyrgyzstan | National | CS | Conv | Contacts of PLHIV | 300ǂ | 3.0 |
| Djumagulova, 2016 [13] | 2012 | Kyrgyzstan | National | CS | Conv | Contacts of PLHIV | 300ǂ | 10.0 |
| Djumagulova, 2016 [13] | 2013 | Kyrgyzstan | National | CS | Conv | Contacts of PLHIV | 300ǂ | 15.0 |
| Djumagulova, 2016 [13] | 2014 | Kyrgyzstan | National | CS | Conv | Contacts of PLHIV | 300ǂ | 11.0 |
| Djumagulova, 2016 [13] | 2014 | Kyrgyzstan | National | CS | Conv | Contacts of PLHIV | 300ǂ | 13.5 |
| Djumagulova, 2016 [13] | 2013 | Kyrgyzstan | National | CS | Conv | MSM | 300ǂ | 0.0 |
| Djumagulova, 2016 [13] | 2014 | Kyrgyzstan | National | CS | Conv | MSM | 300ǂ | 0.0 |
| Djumagulova, 2016 [13] | 2013 | Kyrgyzstan | National | CS | Conv | Patients with STDs | 300ǂ | 3.0 |
| Djumagulova, 2016 [13] | 2014 | Kyrgyzstan | National | CS | Conv | Patients with STDs | 300ǂ | 2.7 |
| Djumagulova, 2016 [13] | 2015 | Kyrgyzstan | National | CS | Conv | Patients with STDs | 300ǂ | 5.8 |
| Djumagulova, 2016 [13] | 2013 | Kyrgyzstan | National | CS | Conv | Sex workers | 300ǂ | 7.0 |
| Djumagulova, 2016 [13] | 2014 | Kyrgyzstan | National | CS | Conv | Sex workers | 300ǂ | 0.0 |
| Azbel, 2016 [15] | 2016 | Kyrgyzstan | Prison | CS | Conv | Prisoners | 368 | 42.4 |
| Ganina, 2016 [16] | 2005 | Kazakhstan | National | CS | Conv | Sex workers | 300ǂ | 16.9 |
| Ganina, 2016 [16] | 2006 | Kazakhstan | National | CS | Conv | Sex workers | 300ǂ | 17.3 |
| Ganina, 2016 [16] | 2007 | Kazakhstan | National | CS | Conv | Sex workers | 300ǂ | 19.2 |
| Ganina, 2016 [16] | 2008 | Kazakhstan | National | CS | Conv | Sex workers | 300ǂ | 12.3 |
| Ganina, 2016 [16] | 2009 | Kazakhstan | National | CS | Conv | Sex workers | 300ǂ | 11.2 |
| Ganina, 2016 [16] | 2010 | Kazakhstan | National | CS | Conv | Sex workers | 300ǂ | 12.4 |
| Ganina, 2016 [16] | 2011 | Kazakhstan | National | CS | Conv | Sex workers | 300ǂ | 13.4 |
| Ganina, 2016 [16] | 2012 | Kazakhstan | National | CS | Conv | Sex workers | 300ǂ | 8.5 |
| Ganina, 2016 [16] | 2013 | Kazakhstan | National | CS | Conv | Sex workers | 300ǂ | 7.2 |
| Ganina, 2016 [16] | 2015 | Kazakhstan | National | CS | Conv | Sex workers | 300ǂ | 7.3 |
| Ganina, 2016 [16] | 2005 | Kazakhstan | National | CS | Conv | Male prisoners | 300ǂ | 39.0 |
| Ganina, 2016 [16] | 2005 | Kazakhstan | National | CS | Conv | Female prisoners | 300ǂ | 33.0 |
| Ganina, 2016 [16] | 2006 | Kazakhstan | National | CS | Conv | Male prisoners | 300ǂ | 40.0 |
| Ganina, 2016 [16] | 2006 | Kazakhstan | National | CS | Conv | Female prisoners | 300ǂ | 38.0 |
| Ganina, 2016 [16] | 2007 | Kazakhstan | National | CS | Conv | Male prisoners | 300ǂ | 43.0 |
| Ganina, 2016 [16] | 2007 | Kazakhstan | National | CS | Conv | Female prisoners | 300ǂ | 42.0 |
| Ganina, 2016 [16] | 2008 | Kazakhstan | National | CS | Conv | Male prisoners | 300ǂ | 43.0 |
| Ganina, 2016 [16] | 2008 | Kazakhstan | National | CS | Conv | Female prisoners | 300ǂ | 40.0 |
| Ganina, 2016 [16] | 2009 | Kazakhstan | National | CS | Conv | Male prisoners | 300ǂ | 42.0 |
| Ganina, 2016 [16] | 2009 | Kazakhstan | National | CS | Conv | Female prisoners | 300ǂ | 50.0 |
| Ganina, 2016 [16] | 2010 | Kazakhstan | National | CS | Conv | Male prisoners | 300ǂ | 42.0 |
| Ganina, 2016 [16] | 2010 | Kazakhstan | National | CS | Conv | Female prisoners | 300ǂ | 37.0 |
| Ganina, 2016 [16] | 2011 | Kazakhstan | National | CS | Conv | Male prisoners | 300ǂ | 40.0 |
| Ganina, 2016 [16] | 2011 | Kazakhstan | National | CS | Conv | Female prisoners | 300ǂ | 32.0 |
| Ganina, 2016 [16] | 2012 | Kazakhstan | National | CS | Conv | Male prisoners | 300ǂ | 36.0 |
| Ganina, 2016 [16] | 2012 | Kazakhstan | National | CS | Conv | Female prisoners | 300ǂ | 31.0 |
| Ganina, 2016 [16] | 2013 | Kazakhstan | National | CS | Conv | Male prisoners | 300ǂ | 32.0 |
| Ganina, 2016 [16] | 2013 | Kazakhstan | National | CS | Conv | Female prisoners | 300ǂ | 29.0 |
| Ganina, 2016 [16] | 2014 | Kazakhstan | National | CS | Conv | Male prisoners | 300ǂ | 30.0 |
| Ganina, 2016 [16] | 2014 | Kazakhstan | National | CS | Conv | Female prisoners | 300ǂ | 29.0 |
| Ruzibakiev , 2001 [2] | 1999-2000 | Uzbekistan | Community | CS | SRS | Sex workers | 87 | 9.2 |
| Kurbanov, 2003 [17] | 2001 | Uzbekistan | Clinical | CS | Conv | Tuberculosis patients | 208 | 18.8 |
| Mun, 2006 [18] | 2005 | Uzbekistan | National | CS | Conv | Sex workers | 1,364 | 12.8 |
| Sentinel surveillance, 2004 [19] | 2004 | Uzbekistan | National | CS | Conv | Sex workers | 315 | 11.0 |

Abbreviations: Conv, convenience; CS, cross-sectional; NS, not specified; SRS, simple random sampling; PLHIV, people living with human immunodeficiency virus; RCT, randomized controlled trial; SBS, snowball sampling; HCW, healthcare workers; MSM, men who have sex with men; CC, case-control study; STDs, sexually transmitted diseases; PWID, people who inject drugs.

^a^Prevalence figures are as reported in the original reports, but rounded to one decimal place, provided the prevalence figure was over 1%.

^b^In randomized controlled trials the extracted HCV prevalence measure was the cross-sectional baseline HCV prevalence measure.

**ǂ**Study did not report sample size. The included sample size was imputed based on the median sample size of all studies that reported a sample size.

¥Study performed on immigrants from Central Asia.

**Figure S3.** Forest plot of studies reporting hepatitis C virus (HCV) prevalence among the general population in Central Asia (CA).


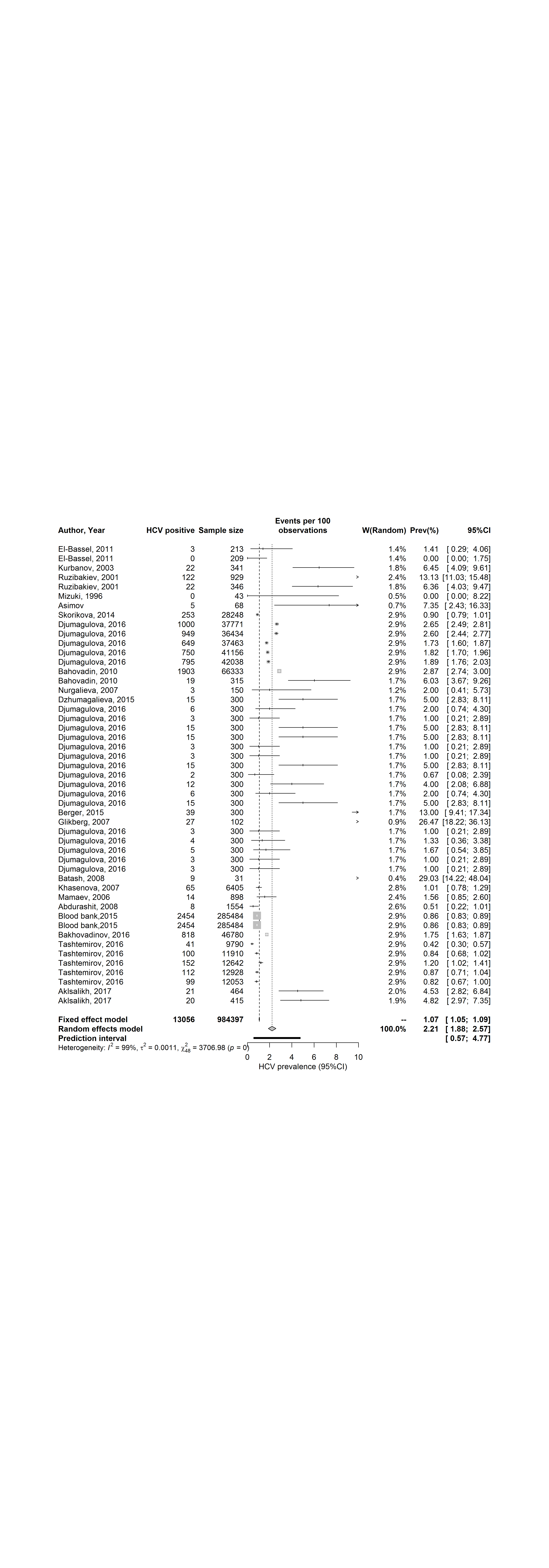


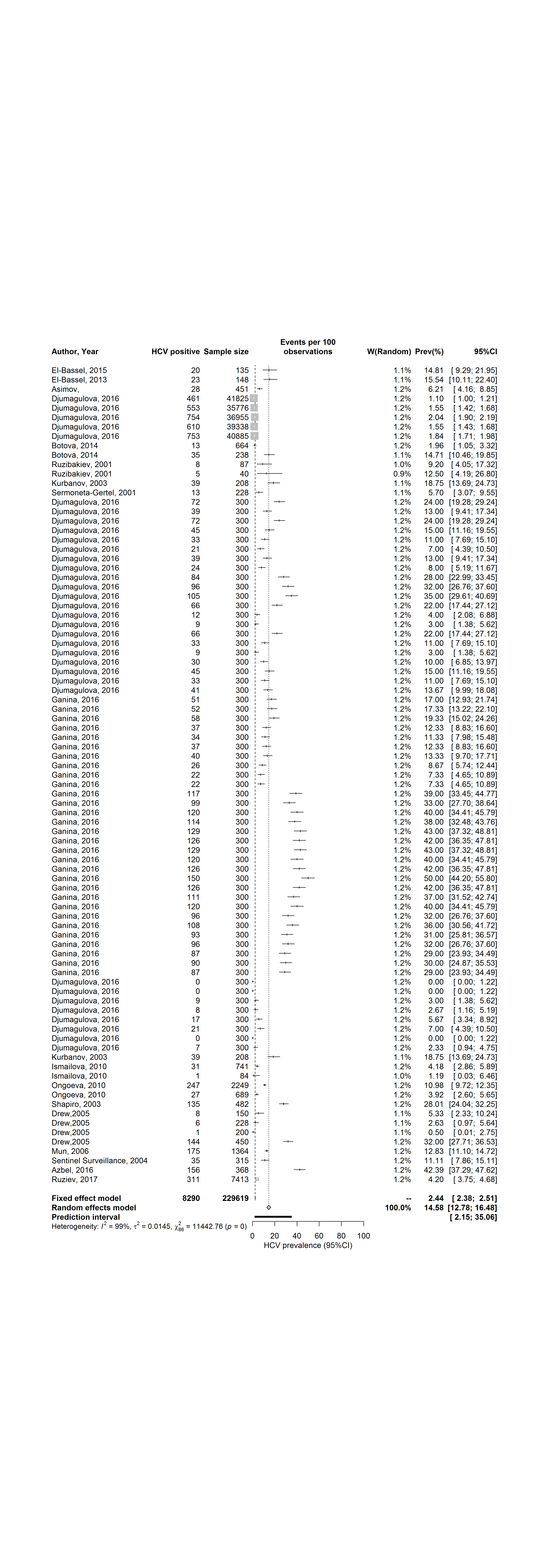
**Figure S4.** Forest plot of studies reporting hepatitis C virus (HCV) prevalence among intermediate risk populations in Central Asia (CA).

**Figure S5.** Forest plot of studies reporting hepatitis C virus (HCV) prevalence among non-specific clinical populations in Central Asia (CA).


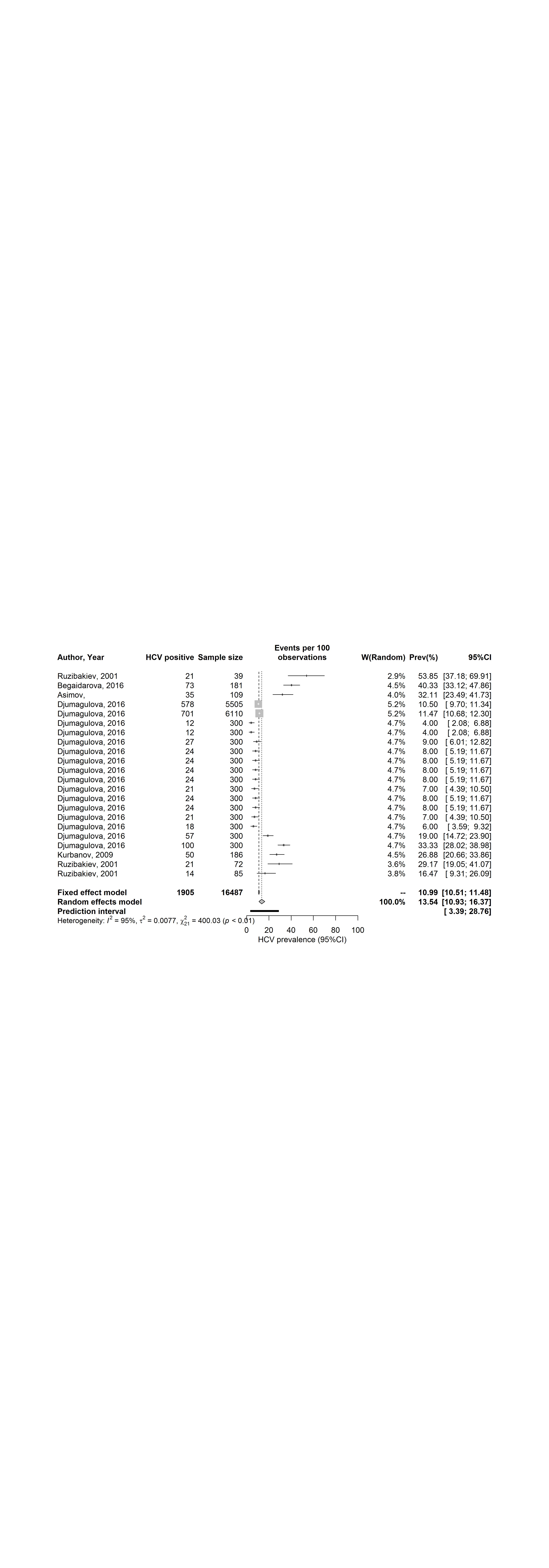


**Figure S6.** Forest plot of studies reporting hepatitis C virus (HCV) prevalence among populations with liver-related conditions in Central Asia (CA).


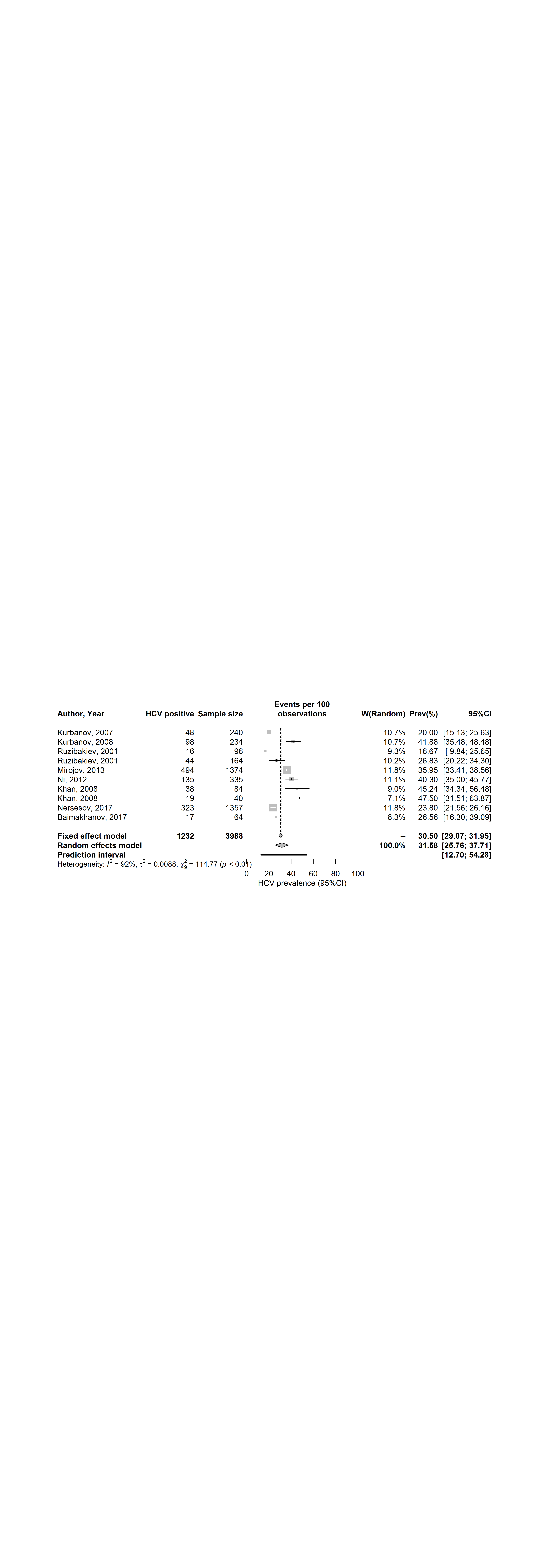


**Figure S7.** Forest plot of studies reporting hepatitis C virus (HCV) prevalence among people who inject drugs (PWID) in Central Asia (CA).


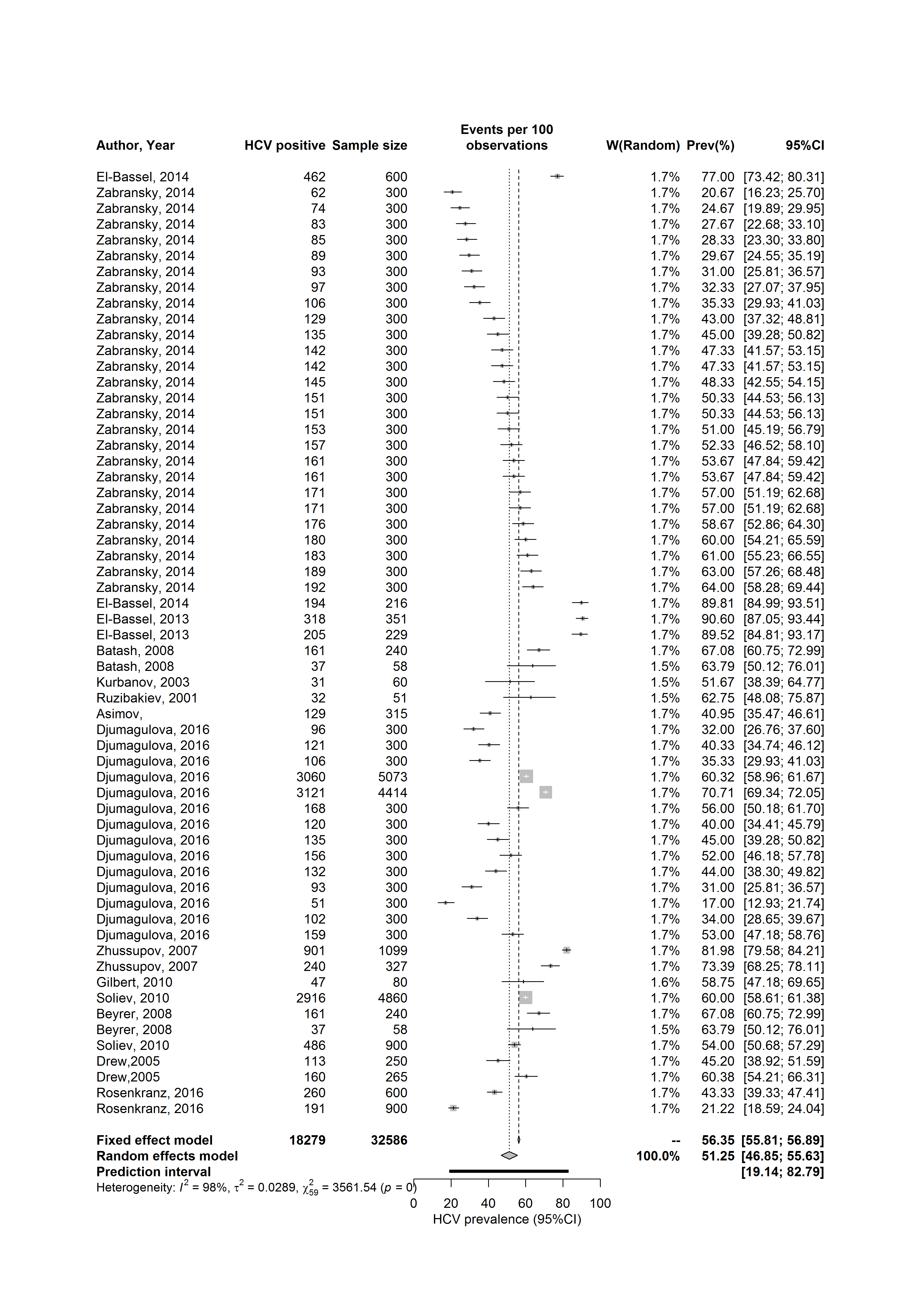


**Figure S8.** A. Map of data availability (number of HCV prevalence studies) across countries of Central Asia (CA). B. Map of the pooled mean HCV prevalence in the general population across countries of Central Asia (CA).

A.

B.


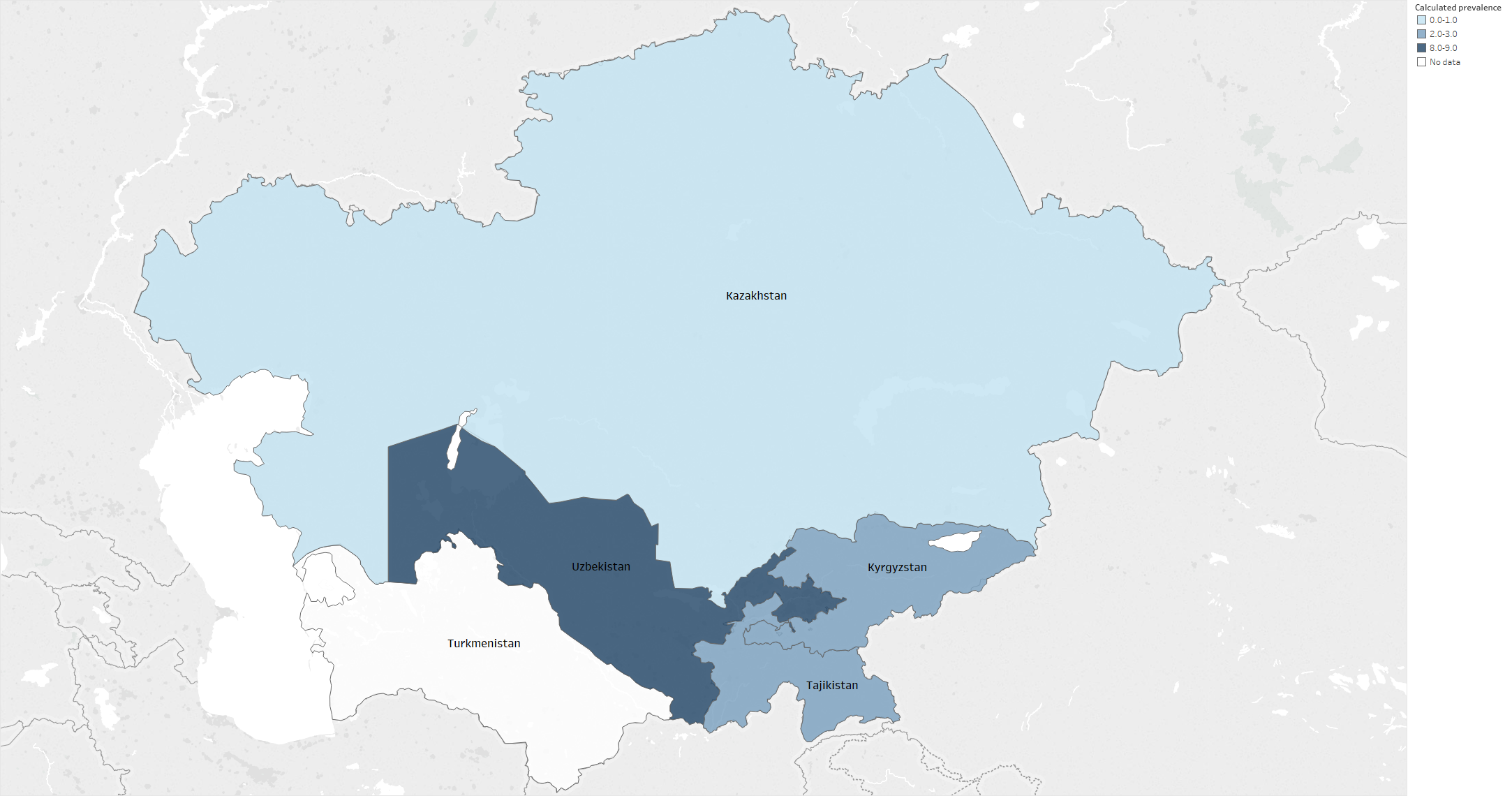

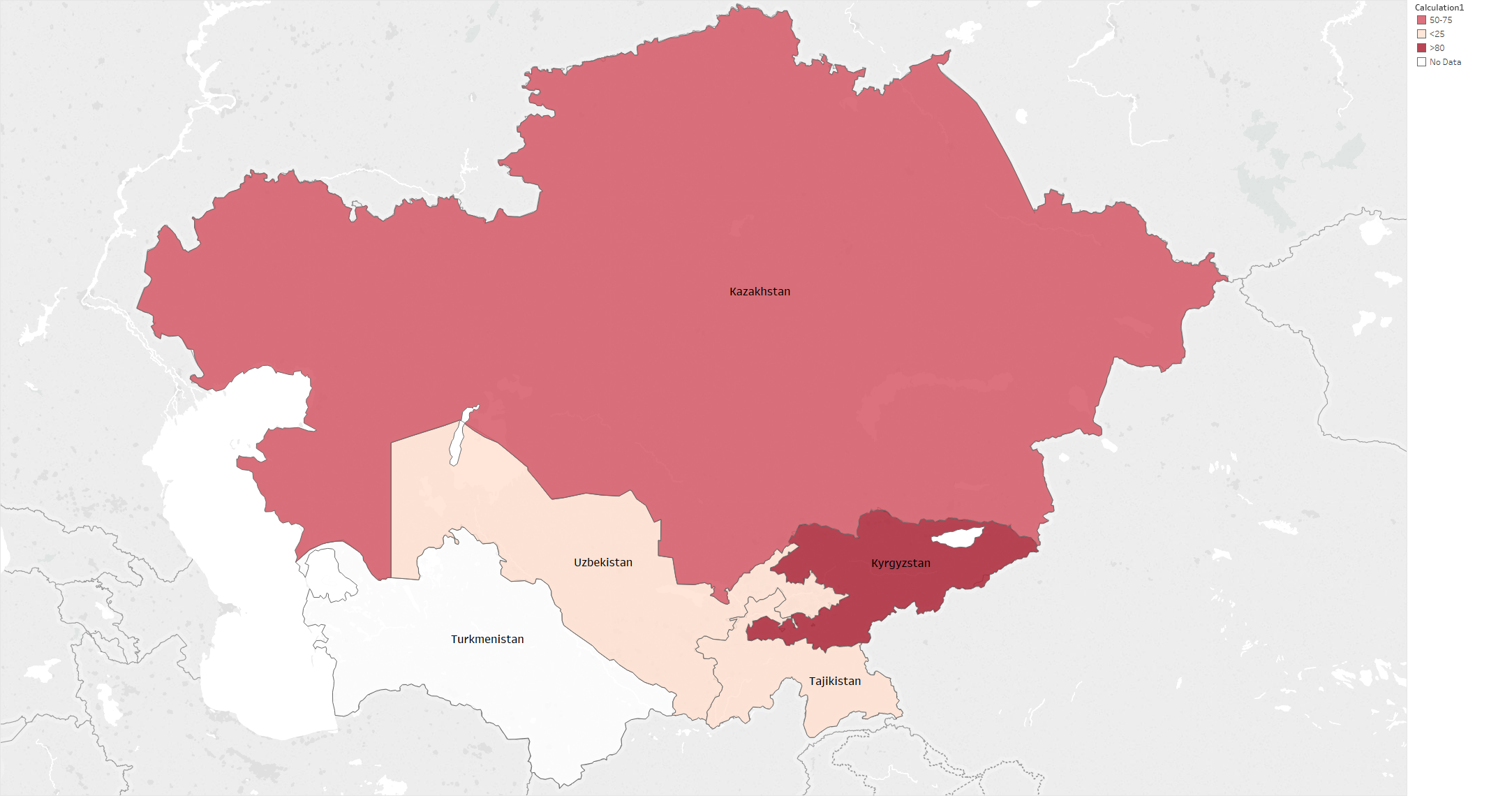

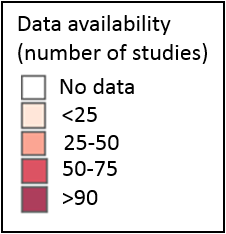

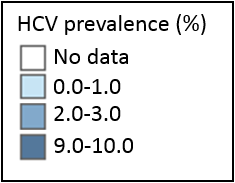


**Table S3.** Comparison of the estimated pooled mean hepatitis C virus (HCV) prevalence in risk populations in Central Asia (CA) using the Freeman-Tukey type arcsine square-root transformation versus the generalized linear mixed models (GLMM).

| Risk population | Studies | Samples | Pooled mean HCV prevalence using the Freeman-Tukey type arcsine square-root transformation | | Pooled mean HCV prevalence using the generalized linear mixed models | |
| --- | --- | --- | --- | --- | --- | --- |
|  | **Total**  **n** | **Total**  **N** | **Random-effects models**  **(95% CI)** | **Fixed-effects models (95% CI)** | **Random-effects models**  **(95% CI)** | **Fixed-effects models (95% CI)** |
| General population | 49 | 984,397 | 2.2% (1.9-2.6) | 1.1 (1.1-1.1) | 2.1 (1.6-2.9) | 1.3 (1.3-1.3) |
| Populations at intermediate risk | 87 | 229,619 | 14.6% (12.8-16.5) | 2.44 (2.4-2.5) | 11.5 (8.9-14.7) | 3.6 (3.5-3.7) |
| Non-specific clinical populations | 22 | 16,487 | 13.5% (10.9-16.4) | 11.0 (10.5-11.5) | 12.7 (9.1-17.6) | 11.6 (11.1-12.1) |
| Populations with liver-related conditions | 10 | 3,988 | 31.6% (25.8-37.7) | 30.5 (29.1-32.0) | 31.4 (25.3-38.1) | 30.9 (29.5-32.3) |
| People who inject drugs | 60 | 32,586 | 51.3% (46.9-55.6) | 56.4 (55.8-56.9) | 51.53 (46.5-56.5) | 56.1 (55.6-55.6) |

**Table S4.** Meta-analyses for hepatitis C virus (HCV) prevalence in Central Asia (CA) among the general population, after excluding blood donor data.

|  | **Studies** | **Samples** | **Prevalence** | | **Pooled HCV prevalence** | | **Heterogeneity measures** | | |
| --- | --- | --- | --- | --- | --- | --- | --- | --- | --- |
|  | **Total n** | **Total N** | **Range (%)**¥ | **Median (%)** | **Mean (%)** | **95% CI** | **Q (p-value)**ª | **I² (confidence limits)^b^** | **Prediction interval (%)^c^** |
| Kazakhstan | 6 | 7,320 | 0.0-5.1 | 1.2 | 1.2 | 0.2-2.7 | 24.9 (p<0.01) | 79.9% (56.3-90.7%) | 0.0-8.0 |
| Kyrgyzstan | 17 | 5,698 | 0.7-5.0 | 1.6 | 2.1 | 1.4-2.8 | 53.5 (p<0.01) | 70.1% (50.9-81.8%) | 0.2-5.6 |
| Tajikistan | 3 | 2,284 | 0.5-6.0 | 4.8 | 3.2 | 0.2-8.9 | 52.5 (p<0.01) | 96.2% (91.9-98.2%) | 0.0-100 |
| Uzbekistan | 4 | 191 | 4.5-29.0 | 13.1 | 12.0 | 6.2-19.4 | 39.8 (p<0.01) | 92.5% (83.9-96.5%) | 0.0-53.3 |
| All countries | 30 | 17,026 | 0.0-29.0 | 1.8 | 2.8 | 1.7-4.0 | 463.0 (p<0.01) | 93.7% (92.1-95.1%) | 0.0-11.9 |

Abbreviations: CI, confidence interval

ªQ: Cochran Q statistic assesses if heterogeneity is present in HCV prevalence estimates.

**^b^**I²: Assesses the percentage of between-study variation that is due to true differences in HCV prevalence estimates across studies rather than chance.

**^c^**Prediction interval: Estimates the 95% interval in which the true HCV prevalence in a new HCV study will lie.

¥This range is for all studies included in the meta-analyses database and covers the range of HCV prevalence across not only main HCV prevalence measures, but also across all strata.

**Table S5.** Frequency, distribution and Shannon Diversity Index of identified hepatitis C virus (HCV) genotypes across countries in Central Asia (CA).

| Country | Kazakhstan | Kyrgyzstan | Tajikistan | Turkmenistan | Uzbekistan | Central Asia |
| --- | --- | --- | --- | --- | --- | --- |
|  | n (%) | n (%) | n (%) | n (%) | n (%) | n (%) |
| Genotype 1 | 18 (24.7%) | - | 44 (84.6%) | - | 139 (54.1%) | 201 (52.6%) |
| Genotype 2 | 19 (26.0%) | - | 4 (7.7%) | - | 13 (5.0%) | 36 (9.4%) |
| Genotype 3 | 36 (49.3%) | - | 4 (7.7%) | - | 105 (40.9%) | 145 (38.0%) |
| Genotype 4 | 0 | - | 0 | - | 0 | 0 |
| Genotype 5 | 0 | - | 0 | - | 0 | 0 |
| Genotype 6 | 0 | - | 0 | - | 0 | 0 |
| Genotype 7 | 0 | - | 0 | - | 0 | 0 |
| *Shannon Diversity Index (H)* | 1.04 | - | 0.54 | - | 0.85 | 0.93 |
| *Index relative to total possible diversity* | 53.66% | - | 27.54% | - | 43.63% | 47.70% |

**Table S6.** Summary of precision and risk of bias assessment (ROB) for hepatitis C virus (HCV) prevalence measures in Central Asia (CA).

| **Quality assessment** | **HCV prevalence** | |
| --- | --- | --- |
|  | **n** | **%** |
| **Precision of estimates** |  |  |
| High precision | 197 | 94.7 |
| Low precision | 11 | 5.3 |
| **Risk of bias quality domains** |  |  |
| **HCV ascertainment** |  |  |
| Low risk of bias | 206 | 99.0 |
| High risk of bias | 2 | 1.0 |
| **Sampling methodology** |  |  |
| Low risk of bias | 79 | 38.0 |
| High risk of bias | 108 | 51.9 |
| Unclear* | 21 | 10.1 |
| **Response rate** |  |  |
| Low risk of bias | 179 | 86.1 |
| High risk of bias | 1 | 0.5 |
| Unclear^*^ | 28 | 13.4 |
| **Total studies where risk of bias assessment was possible** | **208** | **100** |
| **Summary of risk of bias assessment** |  |  |
| **Low risk of bias** |  |  |
| In at least one quality domain | 208 | 100 |
| In at least two quality domains | 130 | 65.0 |
| In all three quality domains | 28 | 13.4 |
| **High risk of bias** |  |  |
| In at least one quality domain | 180 | 86.5 |
| In at least two quality domains | 0 | 0 |
| In all three quality domains | 0 | 0 |
| **Total studies where risk of bias assessment was possible** | **208** | **100** |
| **Total studies** | **208** | **100** |

^*^Studies with missing information for any of the domains were classified as having unclear risk of bias for that specific domain.

**References**

1. Moher D, Liberati A, Tetzlaff J, Altman DG. Preferred reporting items for systematic reviews and meta-analyses: the PRISMA statement. Annals of internal medicine. 2009;151(4):264-9.

2. Ruzibakiev R, Kato H, Ueda R, Yuldasheva N, Hegay T, Avazova D, et al. Risk factors and seroprevalence of hepatitis B virus, hepatitis C virus, and human immunodeficiency virus infection in uzbekistan. Intervirology. 2001;44(6):327-32. Epub 2002/01/24. PubMed PMID: 11805437.

3. Deryabina A, Patnaik P, Gwynn C, El-Sadr WM. Sexual transmission of HIV and possible underreporting of drug use in Kazakhstan. Topics in Antiviral Medicine. 2015;23:474. PubMed PMID: 72119923.

4. El-Bassel N, Gilbert L, Terlikbayeva A, Beyrer C, Wu E, Shaw SA, et al. HIV risks among injecting and non-injecting female partners of men who inject drugs in Almaty, Kazakhstan: implications for HIV prevention, research, and policy. Int J Drug Policy. 2014;25(6):1195-203. Epub 2014/02/22. doi: 10.1016/j.drugpo.2013.11.009. PubMed PMID: 24556208.

5. El-Bassel N, Gilbert L, Terlikbayeva A, Wu E, Beyrer C, Shaw S, et al. HIV among injection drug users and their intimate partners in Almaty, Kazakhstan. AIDS Behav. 2013;17(7):2490-500. Epub 2013/04/25. doi: 10.1007/s10461-013-0484-2. PubMed PMID: 23612942.

6. Ismailova A. HIV-Infection Situation Analysis among MSM in Central Asia, 2010. Presented at the regional conference: HIV infection epidemic in Central Asia: further development of epidemiological surveillance; Almaty, May 18–19, 2010.

7. Ongoeva D. HIV-infection epidemiological analysis among sex workers in Central Asia. Presented at the regional conference: HIV infection epidemic in Central Asia: further development of epidemiological surveillance; Almaty, May 18–19, 2010.

8. Sermoneta-Gertel S, Donchin M, Adler R, Baras M, Perlstein T, Manny N, et al. Hepatitis c virus infection in employees of a large university hospital in Israel. Infect Control Hosp Epidemiol. 2001;22(12):754-61. Epub 2002/03/06. doi: 10.1086/501858. PubMed PMID: 11876453.

9. Azimova SM, Dustov A, Tursunov R. Chronic hepatitis "C" in Tajikistan [Russian]. Avicenna Tajik State Medical University. 2015;2(63):82-9.

10. Mehmondustovich RM. RESULTS OF THE TRANSMISSION OF EPIDEMIOLOGICAL SURVEILLANCE FOR THE PREVALENCE OF HIV INFECTION AMONG THE WORKERS OF COMMERCIAL SEX IN TAJIKISTAN. Healthcare in Tajikistan. 2017;2:42-8.

11. Shapiro BM. Report of sentinel surveillance of HIV infection among sex workers in the Chui, Issyk-Kul, and Jalal-Abad regions of the Kyrgyz Republic. The Director-General of the "AIDS". In Russian: Отчет по проведенному дозорному эпиднадзору за ВИЧ-инфекцией среди работников секса в Чуйской, Иссык-Кульской, Жалалабатской областях Кыргызской Республики. Provided by the WHO Country Office Kyrgyzstan. .

12. Drew R, Choudhri Y. Assessment of HIV/AIDS surveillance in the Europe and Eurasia Region. USAID, 2005.

13. Djumagulova A.Ş. IKA. The situation on viral hepatitis in the Kyrgyz Republic [Russian]. 2016.

14. Ботова О. Распространенность вирусных гепатитов В и С среди медицинских работников и больных неинфекционного стационара по результатам определения маркеров в сыворотках крови. Медицина и экология. 2012;(1 (62)).

15. Azbel L, Polonsky M, Wegman M, Shumskaya N, Kurmanalieva A, Asanov A, et al. Intersecting epidemics of HIV, HCV, and syphilis among soon-to-be released prisoners in Kyrgyzstan: implications for prevention and treatment. International Journal of Drug Policy. 2016;37:9-20.

16. Ganina LY, Elizarieva L, Kaspirova A. Report: Overview of the epidemiological situation of HIV in the Republic of Kazakhstan in 2013-2015. Republican Center on Prevention and Control of AIDS.

17. Kurbanov F, Tanaka Y, Sugauchi F, Kato H, Ruzibakiev R, Zalyalieva M, et al. Hepatitis C virus molecular epidemiology in Uzbekistan. J Med Virol. 2003;69(3):367-75. Epub 2003/01/15. doi: 10.1002/jmv.10298. PubMed PMID: 12526047.

18. Mun Y. Results of epidemiological surveillance over HIV infection in pilot oblasts and behavioural survey among SW in 2005. Presented at: Results of HIV sentinel surveillance and behavioural assessment of the risk groups. Tashkent, 25–26 May, 2006.

19. Results of sentinel surveillance among IDUs and sex workers in Uzbekistan in 2004. Presentation given in Tashkent, May 2005. Provided by the WHO Country Office Uzbekistan.
